# Recognition Selectivities of Lasso-Type Pseudo[1]rotaxane Based on a Mono-Ester-Functionalized Pillar[5]arene

**DOI:** 10.3390/molecules24152693

**Published:** 2019-07-24

**Authors:** Wen-Xue Zhang, Lu-Zhi Liu, Wen-Gui Duan, Qing-Qing Zhou, Cui-Guang Ma, Yan Huang

**Affiliations:** 1 School of Chemistry and Chemical Engineering, Guangxi University, Nanning 530004, China; 2 Guangxi Institute of Chinese Traditional Medical & Pharmaceutical Science and Guangxi Key Laboratory of Tradtitional Chinese Medicine Quality Standards, Nanning 530022, China

**Keywords:** pillar[5]arenes, pseudo[1]rotaxane, host–guest behavior, self-assembly

## Abstract

Two types of mono-ester-functionalized pillar[5]arenes, P1 and P2, bearing different side-chain groups, were synthesized. Their host–guest complexation and self-inclusion properties were studied by ^1^H NMR and 2D nuclear overhauser effect spectroscopy (NOESY) NMR measurements. The results showed that the substituents on their phenolic units have a great influence on the self-assembly of both pillar[5]arenes, although they both could form stable pseudo[1]rotaxanes at room temperature. When eight bulky 4-brombutyloxy groups were capped on the cavity, instead of methoxy groups, pseudo[1]rotaxane P1 became less stable and its locked ester group in the inner space of cavity was not as deep as P2, leading to distinctly different host–guest properties between P1 and P2 with 1,6-dibromohexane. Moreover, pillar[5]arene P1 displayed effective molecular recognition toward 1,6-dichlorohexane and 1,2-bromoethane among the guest dihalides. In addition, the self-complex models and stabilities between P1 and P2 were also studied by computational modeling and experimental calculations.

## 1. Introduction

Pseudo[n]rotaxanes (n ≥ 2), a lasso-type of complexes, are two molecules or multiple molecules mechanically interlocked. Due to the characteristic topological structures and great application in molecular switches [1,2,3] and molecular muscles [4,5,6], mechanically interlocked molecules, especially pseudo[1]rotaxanes (one of lasso-type self-inclusion complexes formed by intramolecular interaction) using traditional macrocycles, such as crown ethers and calixarenes, as their own wheels [7,8,9,10], have become a central topic in supramolecular chemistry. Pillar[n]arenes, a new generation of macrocyclic hosts having a pillar architecture and π-rich cavities, have been widely applied in drug delivery [11,12,13,14], chemosensors [15,16,17], materials [18,19,20], and supramolecular polymers [21,22,23]. Now, pillararene-based pseudo[1]rotaxanes have attracted remarkable attention due to their simple interlocked structures and reversible conversion behavior. Cao et al. [24] found that a mono-ester pillar[5]arene could successfully form a stable pseudo[1]rotaxane which exhibited selective complexation with dihalogen alkanes. A similar compound, with an amino group instead of the ester group, also produced a stable pseudo[1]rotaxane by intramolecular hydrogen bonding in a study by Hou [25]. Moreover, Wang et al. [26,27,28] designed three dynamic pseudo[1]rotaxanes by introducing different groups, including the urea, N-Boc, and biotin moieties to the side-chain of pillar[5]arenes, of which some showed a dynamic, slow disassembly process upon adding competitive guests or strong-polar solvents. Recently, Wang et al. [29] reported a mono-alkyl-functionalized pillar[5]arene that could form a stable pseudo[1]rotaxane in a strong-polar solvent dimethyl sulfoxide (DMSO) and showed responsiveness to sodium cations.

Obviously, pseudo[1]rotaxanes of pillar[n]arene-type have an important application in molecular switches and host–guest complexation. Their mono-substituent groups play an essential role in the self-assembly of the formed pseudo[1]rotaxanes. In fact, the host–guest complexation and self-assembly properties of the pillar[5]arenes is affected by the side-chain groups. However, until now, very little has been known about the influence on the self-inclusion complex properties of pseudo[1]rotaxanes by the other unlocked groups on the benzene ring. In particular, the pillar[n]arene-based pseudo[1]rotaxanes of which molecular recognition can be achieved through controlling their unlocked groups have not yet been reported on.

**Scheme 1:**
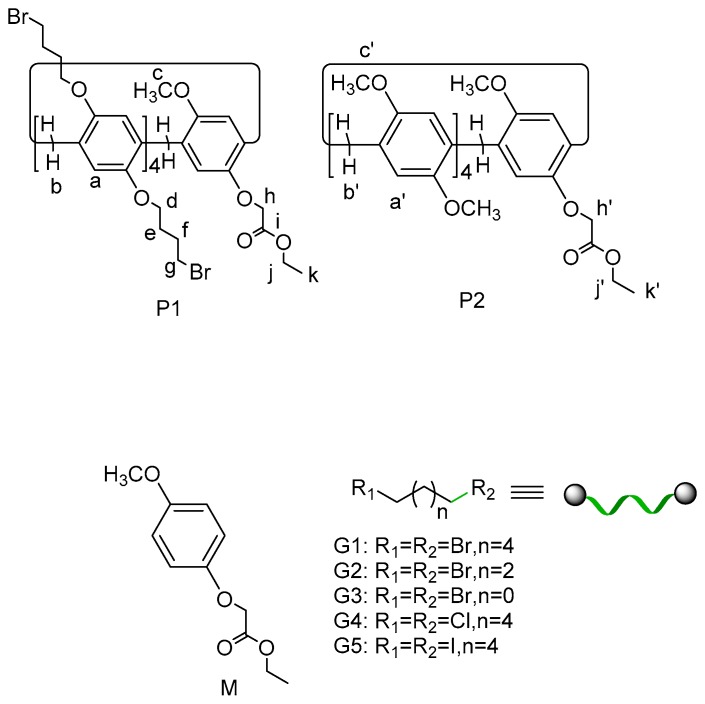
Chemical structures of the hosts and the guests.

In our previous studies [30,31,32,33,34], we found that pillar[5]arene showed good binding properties to linear guests such as α,ω-dihaloalkane. Their complexation behavior with the guest (for instance, with 1,4-dibromobutane) was affected by the different substituents on the rings. Furthermore, in mono-(ω-hydroxyalkoxy)-functionalized pillar[5]arenes, the pillar[5]arene bearing a 6-hydroxyhexyloxy group exhibited a reversible hugging dimer by the intermolecular self-assembly; whereas the pillar[5]arene bearing a short 4-hydroxybutyloxy group did not show such a self-assembly. Thus, we expect that, when an ethyl acetate moiety (as a locked group) is introduced to the side-chain of pillar[5]arene, a stable pseudo[1]rotaxane can be obtained. More importantly, if we used bulky 4-brombutyloxy groups, having a similar-length chain to the ethyl acetate, as other unlocked groups, we could manipulate the stability or self-inclusion behavior, and achieve molecular recognition. In this paper, we synthesized a pillar[5]arene P1 bearing an acetate chain and multi-bromoalkyl chain groups and investigated its self-inclusion behavior and host–guest properties with dihalides such as 1,6-dibromohexane (G1), 1,4-dibromobutane (G2), 1,2-bromoethane (G3), 1,6-dichlorohexane (G4), and 1,6-diiodohexane (G5) (see Scheme 1) by ^1^H NMR and 2D nuclear overhauser effect spectroscopy (2D NOESY) NMR measurements. For comparison, the mono-ester pillar[5]arene P2 [31] was synthesized. In addition, the self-complex models and stabilities between pseudo[1]rotaxane P1 and pseudo[1]rotaxane P2 were also studied by computational modeling and experimental calculations. The syntheses of P1 and P2 are shown in Figure S1.

## 2. Results and Discussion

### 2.1. Self-Assembly Behavior of P1 and P2

**Figure 1:**
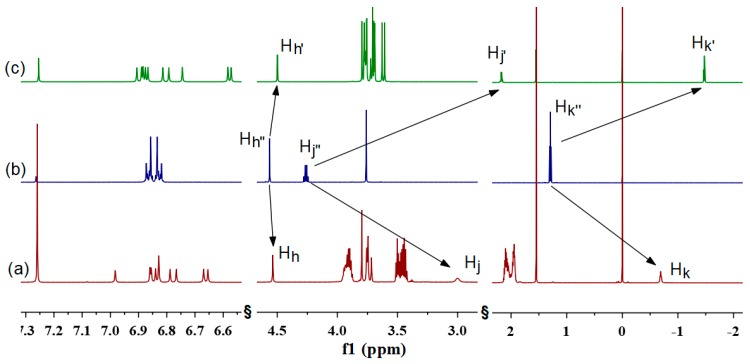
The ^1^H NMR (600 MHz, CDCl_3_, 298 k) spectra of (**a**) P1 (11.16 mM), (**b**) M (11.16 mM), and (**c**) P2 (11.16 mM).

**Scheme 2:**
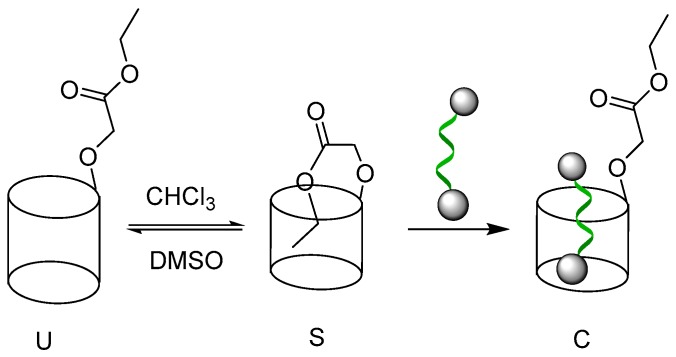
Self-assembly of pilar[5]arenes P1 and P2.

The inclusion behavior of P1 was first studied by ^1^H NMR spectroscopy. As shown in Figure 1, the proton signals of the ethyl acetate chain (H_j_ and H_k_) on pillar[5]arene showed a very substantial upfield shift (Δδ_Hj_ = 1.26 ppm, Δδ_Hk_ = 1.98 ppm) compared to M, indicating that the ethyl acetate moiety was locked in the shielded space of cavity of the pillar[5]arene in CDCl_3_. This result was also consistent with the 2D NOESY NMR spectroscopy of P1, as shown in Figure S15, based on the strong nuclear overhauser effect (NOE) correlation of the proton H_j_ with the protons H_g_ and H_d_, respectively. Meanwhile, correlations between the proton H_k_ and protons H_e_, H_f_, H_g_, and H_d_ could be also observed, which demonstrated that the alkyl chain was included into the inner space of cavity of pillar[5]arene to form a self-inclusion complex. To further confirm the self-assembly model of P1, the ^1^H NMR spectroscopy at variable concentration was measured (see Figure S9). It was found that the self-inclusion structure formed by P1 was very stable at a low concentration (3.72 mM) in CDCl_3_. As the concentration increased, the proton resonances did not exhibit apparent changes, even at a higher concentration of 89.28 mM, suggesting that the self-inclusion complex of P1 was concentration-independent. Thus, a stable pseudo[1]rotaxane of P1 was formed by intramolecular self-assembly in CHCl_3_ solution at room temperature (see Scheme 2).

Similarly, pillar[5]arene P2 also could form a stable pseudo[1]rotaxane in CHCl_3_ solution, according to the obvious upfield chemical shifts of the protons of the ethyl acetate group (see Figure 1c). In contrast to P1, the proton signals of H_h’_, H_j’_, and H_k’_ in P2 showed a larger upfield shift (Figure 1a,c), indicating that the locked ethyl acetate group of P2 in the inner space of cavity was deeper than that in P1. These results showed that the unlocked substituents on their phenolic units had a great influence on the self-inclusion behavior of pseudo[1]rotaxane. When eight bulky 4-brombutyloxy groups were capped on the cavity, instead of methoxy groups, the locked ethyl acetate group of P1 was inhibited from threading into the deeper cavity, leading to a less-stable self-inclusion structure.

When the transformation process between self-inclusion complex (S) and uncomplex (U) occurred in the system (see Scheme 2), the self-assembly behavior such as disassembly rate of pillar[5]arenes could be reflected by ^1^H NMR method (OCH_2_CH_3_) according to Equation (1) due to the shielded and deshielded protons, which were located in the inner and outer electron-rich cavity, respectively. To better understand the influence of solvents on the self-assembly of the pillar[5]arene P1 and P2, we studied their self-inclusion behavior in different ratios of CDCl_3_/DMSO solvents by ^1^H NMR spectroscopy (see Figures S16 and S17). The result showed that partial pseudo[1]rotaxane P1 was disassembled when DMSO-*d*_6_ was added to CDCl_3_. As the DMSO increased, the relative disassembly rate (α) of P1 from S to U was enhanced sharply at first (Δα = 63.62%, DMSO/CDCl_3_ = 0~75%, see Figure S18) and then slowly (Δα = 0.68%, DMSO/CDCl_3_ = 75~90%). When the ratio of DMSO/CDCl_3_ further increased to 90%, the dynamic equilibrium process of S⇌U was almost saturated and the disassembly rate was as high as 64.39%. Similarly, the disassembly behavior of P2 also occurred in DMSO/CDCl_3_ system. Since the lower relative disassembly rate of P2 compared to the P1 system, we can conclude that pseudo[n]rotaxane of P1 is easier to disassemble than P2 in the same ratio of DMSO/CHCl_3_ solvents. It meant that pseudo[n]rotaxane P1 is less stable compared to P2. The disassembly rate α of S⇌U in DMSO/CDCl_3_ system relative to CDCl_3_ was calculated according to Equation (1). Here, δ_S_ and δ are the chemical shifts of self-inclusion complex P1 (H_k_) or P2 (H_k’_) in chloroform and different ratios of DMSO/CDCl_3_, respectively. The δ_U_ are the chemical shifts of uncomplex P1 (H_k_) or P2 (H_k′_), which is close to the chemical shift of the M (H_k′’_)

δ = (1 − α) × δ_S_ + α × δ (1)

### 2.2. Host–Guest Properties

**Figure 2:**
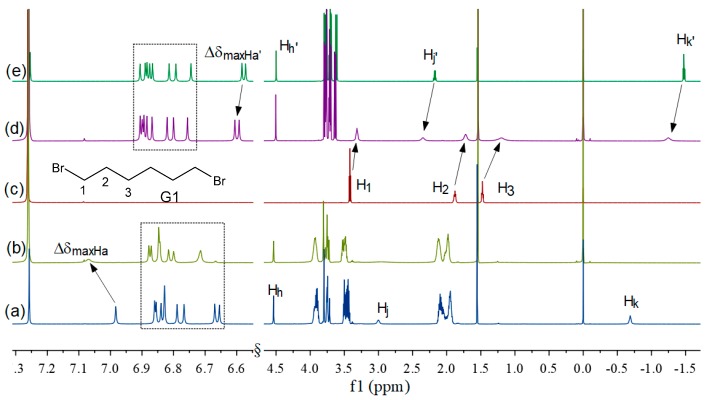
The ^1^H NMR (600 MHz, CDCl_3_, 298 k) spectra of (**a**) P1, (**b**) P1 + 1eq G1 (3.72 mM), (**c**) G1, (**d**) P2 + 1eq G1 (3.72 mM), and (**e**) P2.

The ^1^H NMR spectra of **G1** in CDCl_3_, in the presence and in the absence of approximately 1 equivalent of P2 (3.72 mmol/L), respectively, are shown in Figure 2. The protons in G1 showed a substantial upfield shift (Δδ = 0.10, 0.16, and 0.28 ppm for H_1_, H_2_, and H_3_, respectively), as well as a broadening effect in the presence of P2, compared to that of free G1. Meanwhile, the proton signals H_j’_ and H_k’_ in the acetate group of P2 displayed a downfield displacement (Δδ = 0.18 and 0.23 ppm for H_j’_ and H_k’_, respectively). This result clearly indicated that the inclusion structure was destroyed and the guest G1 was locked in the cavity to form a pseudo[2]rotaxane P2. In contrast, the binding behavior of P1 with G1 was very different. When an equimolar guest G1 was mixed with P1, the protons on the phenyl rings of P1 were shifted to the downfield region. Surprisingly, the protons (H_1_, H_2_, and H_3_) of G1 and the proton signals of the acetate chain (H_j_ and H_k_) all disappeared completely in the ^1^H NMR spectra (−2~10 ppm), possibly due to a remarkable broadening effect (these protons are in intermediate exchange in the NMR time scale, and therefore the signals broaden and seem to disappear in the baseline). It has been suggested that the guest G1 was locked into the cavity, which also agrees with the literature [33,35], such that the protons of the guests could not be observed in the inclusion complexes. And the 1:1 complexation stoichiometry of G1⊂P1 was obtained by the Job’s plots (Figure S14). In addition, we have investigated the host–guest properties of P1 and P2 with G3 by ^1^H NMR spectra (Figure S19). As shown in Table S3, the chemical shift of the guest proton H_1_ in G3⊂P2 system showed substantial upfield shift (∆δ_H1_ = 1.91 ppm), which was larger than the value of the ∆δ_H1_ of 1.36 ppm in G3⊂P1 system, indicating that the complexation of G3⊂P2 system was stronger than that of G3⊂P1. These results showed that the host–guest complexation properties of pseudo[1]rotaxanes were also influenced by alkyl bromide side-chains.

Generally, in the formation of a pseudo[2]rotaxane by pillar[5]arene and a guest, the rotation of the phenolic units around the CH_2_ bridge would be inhibited, resulting in less-shielded protons in the pillar[5]arene in the host–guest system [32]. Thus, the protons of the phenolic units were usually shifted downfield and the chemical shift change value (Δδ = δ_complex_ − δ_uncomplex_) could be used to evaluate the conformational characteristics of pillar[5]arenes. In G1⊂P1 and G1⊂P2 systems, most of the protons of the phenolic units slightly changed, compared to their corresponding pseudo[1]rotaxanes (see Figure 2), indicating that the rotation of the benzene rims had been well-inhibited in P1 and P2, but not immobilized absolutely. This result also further proved that both the pillar[5]arenes P1 and P2 can form a stable pseudo[1]rotaxane, in the absence of **G1**. In addition, according to the smaller downfield shifts (Δδ_maxHa_ = 0.087 ppm and Δδ_maxHa′_= 0.001 ppm) of the phenyl proton, we can demonstrate that the freedom of the phenolic unit rotations in P2 was lower than that in P1. This meant better stability for P2, in agreement with the above experiment.

The visible influence of the self-inclusion properties of P1 by substituents on its phenolic units and its extraordinary host–guest behavior made us interested in its molecule complexation. Thus, the interactions of P1 with G2–G5 were also investigated (see Table S4 and Figures S10–S13). It was found that all guests can form complexes with P1, resulting in pseudo[2]rotaxane structures based on the obvious downfield shift for the protons of phenyl groups (H_a_) and methylene bridges (H_b_). However, the proton signals of the guests and the ethyl acetate chain (H_j_ and H_k_) showed evident differences in the different host–guest systems. Similar to the NMR changes in the G1⊂P1 system, the protons of the guests and the ethyl acetate chain of P1 in the G2⊂P1 and G5⊂P1 systems, were not observed. On the other hand, for 1,2-dibromoethane (G3), when the host–guest complex G3⊂P1 was formed, the protons of G3 showed an obvious upfield shift and the proton H_k_ was shifted downfield. However, the proton H_j_ was not observed in the ^1^H NMR, which was similar to G1⊂P1system. In addition, the binding behavior between 1,6-dichlorohexane (G4) and P1 was also very interesting. After 1eq guest was added to P1 in CHCl_3_, all the guest protons and H_k_ could not be found and the chemical shift of the proton H_j_ was nearly unchanged, which distinguished this system, compared to the other systems, very much. According to the above results, P1 showed molecular recognition toward G3 and G4 among the α,ω-dihalides (G1–G5).

### 2.3. Quantum Chemical Calculations

**Figure 3:**
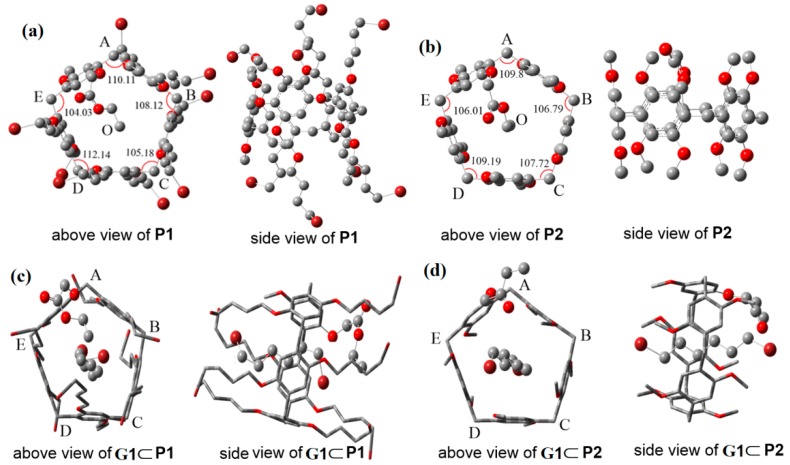
Optimized geometries of the self-inclusion and/or host–guest structures of (**a**) P1, (**b**) P2, (**c**) G1⊂P1, and (**d**) G1⊂P2. (Oxygen atoms are shown in red, bromine atoms are shown in red brown. Note that the H atoms are not presented in the figure for convenience.)

The mono-locked and other unlocked groups on the side-chain of pillar[5]arene played an essential role in determining the self-inclusion and molecular-complex properties of the hosts. In order to further understand the spatial structure, with the goal of designing functional pseudo[1]rotaxanes of pillar[5]arene, density functional theory (DFT) calculations were carried out to optimize the structures of P1 and P2 at the B3LYP/6-31G(d, p) level in CHCl_3_, by employing the Gaussian 09 program package [36]. The optimized pillar[5]arene-based pseudo[1]rotaxane structures are shown in Figure 3, and some selected geometric parameters are listed in Table 1.The cavities of P1 and P2 showed highly regular and symmetric pentagons, which are made up of five para-methylene bridges (A, B, C, D, and E; see Figure 3). However, the five points A, B, C, D, and E were not completely in a plane, based on their inner angles (ψ). The Δψ (ψ_max_ − ψ_min_) value of P1 was 8.11°, larger than that of P2 (3.79°), indicating that the cavity of P1 showed more variation and less stability compared to P2. This result was assisted by the previous analysis and further proved by the direct proof that the energy is 266.357 KJ/moL and 154.608 KJ/moL for P1 and P2, respectively. In addition, all the distances between the locked-CH_3_ group (O) and the para-methylene bridges (A, B, C, D, and E) in P1 were longer than those in P2 except for O–B, which demonstrates that the locked ethyl acetate group of P1 in the inner space of cavity was not deeper than that in P2.

In host–guest systems, after formation of the host–guest complexes, the locked ester groups of P1 and P2 were squeezed out the cavities (see Figure 3c,d). However, the model structures G1⊂P1 and G1⊂P2 were quite different. For G1⊂P1, the locked guest G1 in the cavity was closer to the ester group due to the greater steric hindrance of other 4-brombutyloxy substituents. In addition, the Br⋯CH_2_ (and CH_3_) interaction between G1 and ester occurred in the system. And the CH_2_CH_3_ group of ester, located in the shielded region projected by the cavity, was almost perpendicular to G1, effectively blocking the guest G1 deeper into the cavity from the other side. Thus, the locked guest G1 was significantly different in depth in both sides of the cavity and its chain length on the ester side was shorter than the one on the OCH_3_ group side (see Figure S21, Tables S5 and S6). In contrast, the guest G1 was naturally locked inside the cavity and the ethyl ester group was located at the edge of cavity where was a deshielding area in G1⊂P2 system. Interestingly, the longer length of G1 was on the ester side of cavity, although the ethyl ester was a bulky group compared to OCH_3_. These calculated binding modes were benefit to understand the effective influence for the host–guest complexation performance of pseudo[1]rotaxanes by their unlocked groups.

**Figure 4:**
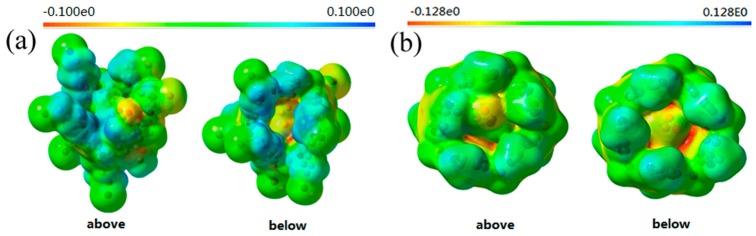
Electrostatic potential (ESP) maps of (**a**) P1 and (**b**) P2.

Two electrostatic potential (ESP) maps each of P1 and P2 (see Figure 4) were also calculated to study their self-inclusion mechanisms, on the basis of previously optimized configurations, using the same theory level in the self-consistent field (SCF) density matrix. For P1, a low electron cloud density (ECD) region was formed along the upper or lower rim of the cavity, due to the effect of the 4-brombutyloxy groups, leading to strong repulsion for –CH_2_CH_3_ of the ethyl acetic group. Thus, pillar[5]arene P1 did not form a self-inclusion structure as easily as pillar[5]arene P2. In addition, when guests were threaded into the cavity to form the pseudo[2]rotaxane P1 complex, they did not only need to overcome the ethyl acetate barrier, but also the low ECD of the alkyl chain groups would be strongly repulsed by the outside of the cavity. These ESP models fairly explicated the higher variation and lesser stability of P1 compared to P2.

## 3. Experimental Section

### 3.1. General

The IR spectra (KBr pellet method) were recorded on a Nicolet iS50 FT-IR spectrometer (Thermo Scientific Co., Ltd., Madison, WI, USA). The ^1^H NMR, ^13^C NMR, and NOESY spectra were recorded in CDCl_3_ solvent on a Bruker Avance III HD 600 MHz spectrometer (Bruker Co., Ltd., Zurich, Switzerland), and the chemical shifts are expressed in ppm (δ) downfield relative to TMS, as an internal standard. The mass spectra were recorded on a TSQ Quantum Access MAX HPLC-MS instrument (Thermo Scientific Co., Ltd., Waltham, MA, USA). The UV–vis absorption spectra were performed on a Shimadzu UV-1800 spectrometer (Shimadzu Corp., Kyoto, Japan). Melting points were determined on a MP420 automatic melting point apparatus (Hanon Instruments Co., Ltd., Jinan, China), and were not corrected. Other reagents were purchased from commercial suppliers and used as received.

### 3.2. Synthesis of P1 and P2

Compounds P1 and P2 were synthesized according to existing procedures [31], and can be found in the Supplementary Information.

P1. White solid. Yield: 19.3%; melting point: 97.1–99.8 °C; IR (KBr) cm^−1^: 2939, 2865 (C–H), 1726.53 (C=O), 1611, 1505, 1470 (Ar–C=C), 1249, 1213, 1047 (Ar–O, C–O); ^1^H-NMR (600 MHz, CDCl_3_): δ = 6.98 (s, 1H, H–a), 6.86 (d, *J* = 2.8 Hz, 2H, H–a), 6.84 (s, 1H, H–a), 6.83 (s, 2H, H–a), 6.79 (s, 1H, H–a), 6.77 (s, 1H, H–a), 6.67 (s, 1H, H–a), 6.65 (s, 1H, H–a), 4.54 (s, 2H, H–h), 3.94–3.88 (m, 16H, H–d), 3.80 (s, 3H, H–c), 3.76–3.72 (m, 10H, H–b), 3.51–3.42 (m, 16H, H–g), 3.00 (d, *J* = 4.9 Hz, 2H, H–j), 2.12–1.94 (m, 32H, H–f, H–e), −0.69 (t, *J* = 7.1 Hz, 3H, H–k); ^13^C-NMR (151 MHz, CDCl_3_): δ = 168.94 (C–i), 150.16, 150.05, 150.01, 149.87, 149.68, 149.66, 149.49, 149.40, 149.30, 149.15, 129.11, 129.00, 128.86, 128.62, 128.52, 128.35, 128.19, 128.05, 127.60, 127.51, 115.90, 115.27, 115.02, 114.81, 114.75, 114.64, 114.62, 114.12, 113.99, 112.97 (C–a), 67.87, 67.82, 67.77, 67.70, 67.45, 67.42, 67.39, 67.34 (C–d), 65.18 (C–h), 60.67 (C–j), 56.36 (C–c), 33.79, 33.76, 33.73, 33.70 (C–b, C–g), 30.98, 30.11, 29.85, 29.83, 29.81, 29.74, 29.64 (C–f), 29.26, 29.06, 28.54, 28.48, 28.43, 28.36, 27.88 (C–e), 11.91 (C–k); ESI–MS m/z: C_72_H_94_Br_8_O_12_: 1806.38 ([M + NH_3_–H]^−^).

P2. White solid. Yield: 30.2%; melting point: 207.3–209.7 °C; IR (KBr) ν/cm^−1^: 3044.81 (Ar–H), 2987.05, 2939.60, 2828.19 (C–H), 1726.53 (C=O), 1615.13, 1503.72, 1466.59 (Ar–C=C), 1214.90, 1047.79 (C–O); ^1^H NMR (600 MHz, CDCl_3_) δ/ppm: 6.91 (s, 1H, Ca–H), 6.89 (d, J = 3.42 Hz, 2H, Ca–H), 6.88 (s, 1H, Ca–H), 6.87 (s, 1H, Ca–H), 6.81 (s, 1H, Ca’–H), 6.79 (s, 1H, Ca’–H), 6.74 (s, 1H, Ca′–H), 6.58 (s, 1H, Ca′–H), 6.57 (s, 1H, Ca′–H), 4.50 (s, 1H, Ch′–H), 3.80 – 3.76 (m, 18H, Cb′–H, Cc′–H), 3.72–3.69 (m, 13H, Cc′–H), 3.63 (s, 3H, Cc′–H), 3.61 (s, 3H, Cc′–H), 2.17 (q, J = 7.02 Hz, 2H, Cj′–H), –1.48 (t, J = 7.02 Hz, 3H, Ck′–H); ^13^C NMR (151 MHz, CDCl_3_) δ/ppm: 169.08 (C–i′), 151.29, 150.81, 150.76, 150.45, 150.43, 150.15, 149.94, 149.92, 149.87, 149.65, 129.32, 129.21, 129.01, 128.65, 128.56, 128.43, 128.03, 127.74, 127.15, 127.12, 115.29, 115.24, 114.17, 114.02, 113.89, 113.42, 113.40, 113.11, 112.68, 112.33 (C–a′), 64.78 (C–h’), 60.56 (C–j′), 56.51, 56.04, 55.95, 55.88, 55.85, 55.71, 55.58, 55.49, 55.20 (C–c′), 31.85, 30.36, 28.99, 28.71, 27.24 (C–b′), 10.77 (C–k′).

## 4. Conclusions

In summary, we synthesized two types of mono-ester-functionalized pillar[5]arens (P1 and P2) bearing different side-chain groups and studied their self-inclusion properties and host–guest complexation. Both of these pillar[5]arenes can form stable pseudo[1]rotaxanes, but their self-inclusion behaviors were greatly affected by the unlocked substituents on their phenolic units. When eight bulky 4-brombutyloxy groups were capped on the cavity, instead of methoxy groups, pseudo[1]rotaxane P1 became less stable and its locked ester group in the inner space of cavity was not as deep as P2, leading to distinctly different host–guest properties between P1 and P2 with 1,6-dibromohexane. In addition, complexation studies revealed that P1 showed good molecular recognition toward the studied guests (G1–G5). The significant information obtained in this study will promote further understanding of the formation of pseudo[1]rotaxanes and the development of their molecular recognition. More importantly, P1, possessing eight 4-brombutyloxy groups, can easily be functionalized by many hosts.

## Figures and Tables

**Table 1:** The distances between the locked-CH_3_ group (O) and the para-methylene bridges (A, B, C, D, and E), and the inner angles of P1and P2.

	P1	P2
Bond lengths (Å)		
O–A	5.91	5.13
O–B	5.53	5.04
O–C	5.27	4.81
O–D	5.62	4.83
O–E	6.33	5.20
Inner angles (°)		
E–A–B	110.11	109.80
A–B–C	108.12	106.79
B–C–D	105.18	107.72
C–D–E	112.14	109.20
D–E–A	104.03	106.01

## Supplementary Materials

Supplementary materials are available online.
