# Supplementary material for: Recognition Selectivities of Lasso-Type Pseudo[1]rotaxane Based on a Mono-Ester-Functionalized Pillar[5]arene
Source: Molecules. 2019 Jul 24;24(15):2693. doi: 10.3390/molecules24152693 (PMC6695583; doi:10.3390/molecules24152693)
Supplement: Supplementary file 1 [file molecules-24-02693-s001.pdf]

Supporting Information

Recognition selectivities of lasso-type pseudo[1]rotaxane based on a monoester-functionalized pillar[5]arene

Wen-Xue Zhang[1], Lu-Zhi Liu[1,*], Wen-Gui Duan[1,*], Qing-Qing Zhou[1], Cui-Guang Ma[1] and Yan Huang[2,*]

[1] School of Chemistry and Chemical Engineering, Guangxi University, Nanning 530004, Guangxi, China

[2] Guangxi Institute of Chinese Traditional Medical & Pharmaceutical Science and Guangxi Key Laboratory of Tradtitional Chinese Medicine Quality Standards, Nanning

530022, Guangxi, China

* Correspondence:Correspondence: llzh068@163.com (L.-Z. Liu), wgduan@gxu.edu.cn (W.-G. Duan), hy2002-2006@163.com (Y. Huang).

S1. Synthesis of P1 and P2 2

S2. ^{1}H NMR spectra of P1 at variant concentrations 6

S3. ^{1}H NMR complexation analysis of P1 with G2-G5 7

S4. UV-Vis absorption of complexation between P1 and G1 9

S5. The 2D NOESY NMR spectrum of pseudo[1]rotaxane P1. 9

S6. The ^{1}H NMR exchange experiments of P1 and P2. 10

S7. The calculation of relative disassembly rate 11

S8. The ^{1}H NMR spectra of the complexation of P1 and P2 with G3 12

S9. The process of Gibbs free energy 14

S10. The Optimized structures 15

S11. Coordinates of Optimized Structures P1, P2, G1⊂P1 and G1⊂P2 16

References: 49

S1. Synthesis of P1 and P2

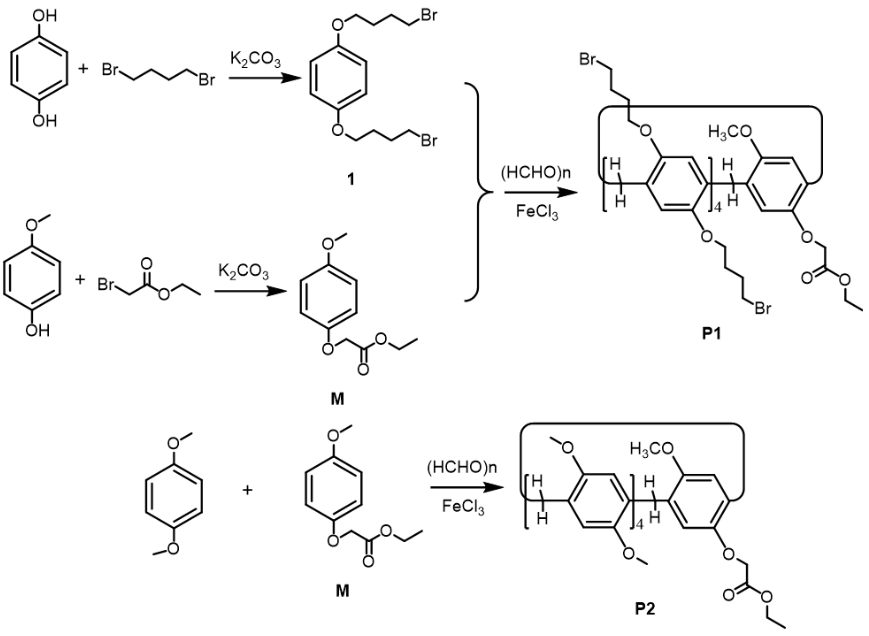

Figure S1. Synthesis of P1 and P2

Compound P1[S3]

Compounds **1**[S1] and M[S2] were prepared according to previous literatures. P1 was prepared by the reaction of compound **1** (69.1 mg, 0.33 mmol), compound M (500 mg, 1.32 mmol), and paraformaldehyde (118.50 mg, 3.95 mmol) in CH_2Cl_2 (150 mL). Then anhydrous ferric chloride (34.14 mg, 0.21 mmol) was added to the solution and the mixture was stirred at room temperature for 3 h in a nitrogen atmosphere. The reaction was quenched by the addition of deionized water (100 mL) and the product was extracted with CH_2Cl_2 (3 × 40 mL). The organic phase was collected, concentrated under reduced pressure and then subjected to column chromatography (silica gel, DCM/PE=2/1, v/v) to give the final product P1 as white solid (19.3%). ^{1}H-NMR (600 MHz, $CDCl_3$): δ = 6.98 (s, 1H, H–a), 6.86 (d, *J* = 2.8 Hz, 2H, H–a), 6.84 (s, 1H, H–a), 6.83 (s, 2H, H–a), 6.79 (s, 1H, H–a), 6.77 (s, 1H, H–a), 6.67 (s, 1H, H–a), 6.65 (s, 1H, H–a), 4.54 (s, 2H, H–h), 3.94–3.88 (m, 16H, H–d), 3.80 (s, 3H, H–c), 3.76–3.72 (m, 10H, H–b), 3.51–3.42 (m, 16H, H–g), 3.00 (d, *J* = 4.9 Hz, 2H, H–j), 2.12–1.94 (m, 32H, H–f, H–e), -0.69 (t, *J* = 7.1 Hz, 3H, H–k); ^{13}C-NMR (151 MHz, $CDCl_3$): δ = 168.94 (C–i), 150.16, 150.05, 150.01, 149.87, 149.68, 149.66, 149.49, 149.40, 149.30, 149.15, 129.11, 129.00, 128.86, 128.62, 128.52, 128.35, 128.19, 128.05, 127.60, 127.51, 115.90, 115.27, 115.02, 114.81, 114.75, 114.64, 114.62, 114.12, 113.99, 112.97 (C–a), 67.87, 67.82, 67.77, 67.70, 67.45, 67.42, 67.39, 67.34 (C–d), 65.18 (C–h), 60.67 (C–j), 56.36 (C–c), 33.79, 33.76, 33.73, 33.70 (C–b ,C–g), 30.98, 30.11, 29.85, 29.83, 29.81, 29.74, 29.64 (C–f), 29.26, 29.06, 28.54, 28.48, 28.43, 28.36, 27.88 (C–e), 11.91 (C–k); ESI–MS m/z: $C_{72}H_{94}Br_8O_{12}$: 1806.38 ($[M + NH_3 – H]^-$).

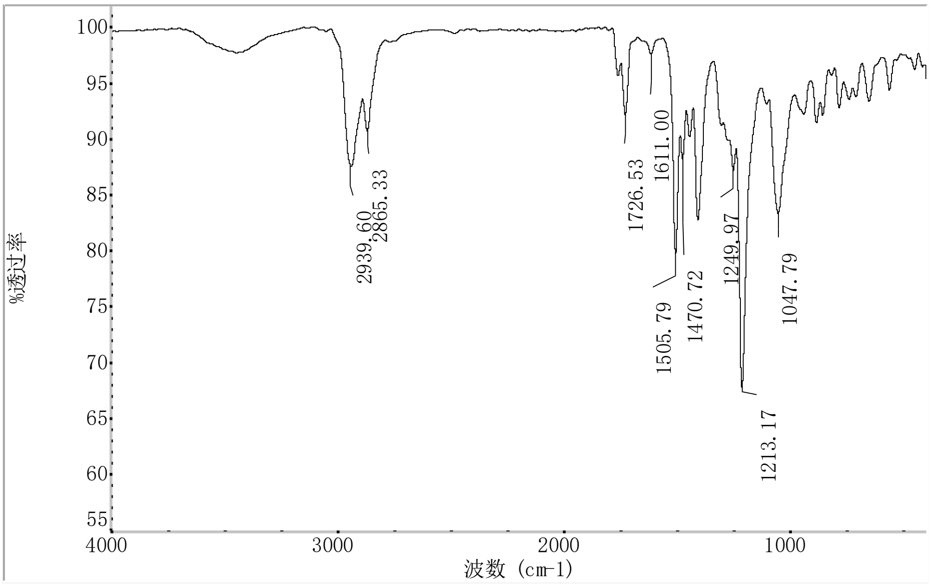

Figure S2. FT-IR spectrum of P1.

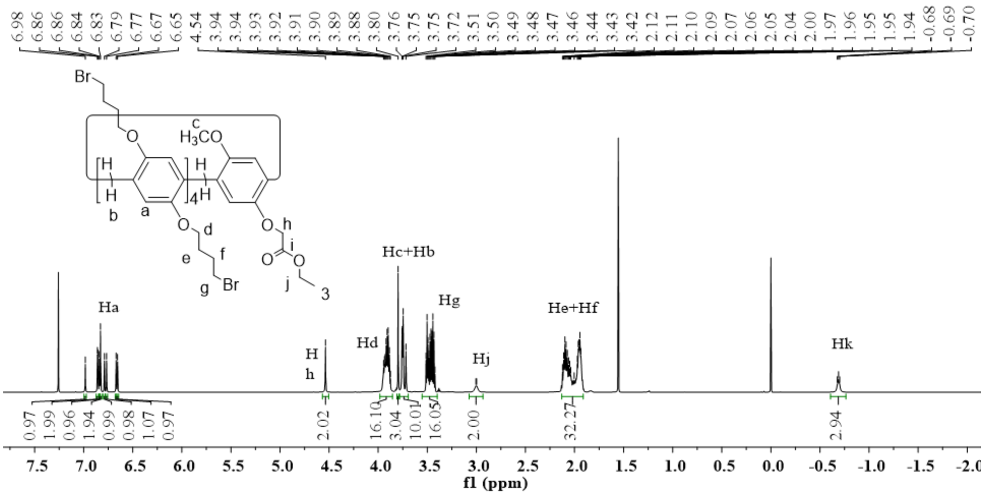

Figure S3. ^{1}H-NMR spectrum of P1 in $CDCl_3$.

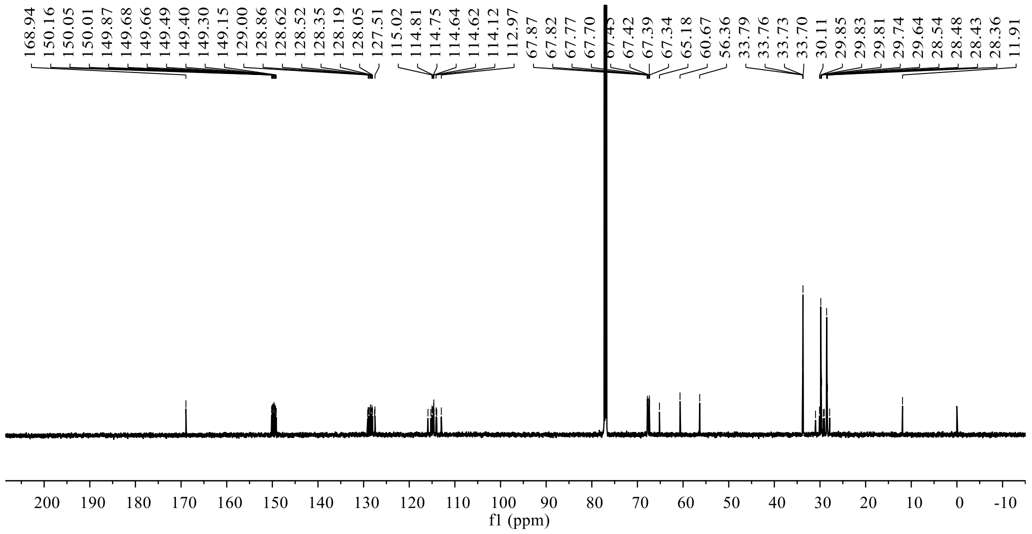

Figure S4. ^{13}C-NMR spectrum of P1 in $CDCl_3$.

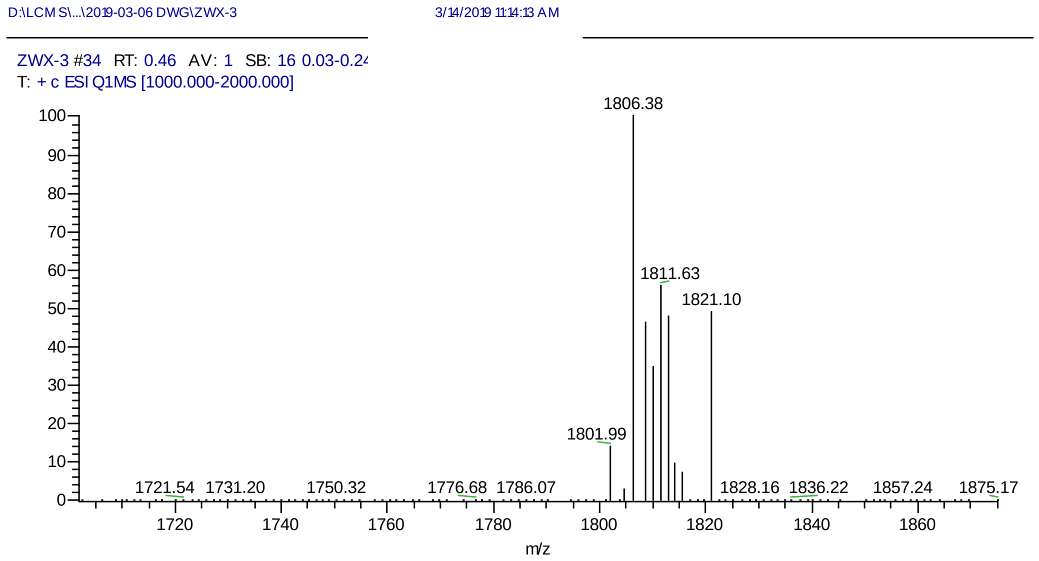

Figure S5. ESI-MS spectrum of P1.

Compound P2[S3]

P2 was prepared by the reaction of 1, 4-dimethoxybenzene (2.63 g, 19.03 mmol), compound M (500 mg, 2.38 mmol), and paraformaldehyde (1.71 g, 57.10 mmol) in CH_2Cl_2 (200 mL). Then anhydrous ferric chloride (401.2 mg, 2.47 mmol) was added to the solution and the mixture was stirred at room temperature for 3 h in a nitrogen atmosphere. The reaction was quenched by the addition of deionized water (150 mL) and the product was extracted with CH_2Cl_2 (3 × 50 mL). The organic phase was collected, concentrated under reduced pressure and the crude product was purified by column chromatography (silica gel, EA/PE=1/15, v/v) to give the final product P2 as white solid (30.2%). ^{1}H NMR (600 MHz, CDCl3) δ/ppm: 6.91 (s, 1H, Ca-H), 6.89 (d, J = 3.42 Hz,

2H, Ca-H), 6.88 (s, 1H, Ca-H), 6.87 (s, 1H, Ca-H), 6.81 (s, 1H, Ca'-H), 6.79 (s, 1H, Ca'-H), 6.74 (s, 1H, Ca'-H), 6.58 (s, 1H, Ca'-H), 6.57 (s, 1H, Ca'-H), 4.50 (s, 1H, Ch'-H), 3.80 – 3.76 (m, 18H, Cb'-H, Cc'-H), 3.72 – 3.69 (m, 13H, Cc'-H), 3.63 (s, 3H, Cc'-H), 3.61 (s, 3H, Cc'-H), 2.17 (q, J = 7.02 Hz, 2H, Cj'-H), –1.48 (t, J = 7.02 Hz, 3H, Ck'-H); ^{13}C NMR (151 MHz, CDCl3) δ/ppm: 169.08 (C-i'), 151.29, 150.81, 150.76, 150.45, 150.43, 150.15, 149.94, 149.92, 149.87, 149.65, 129.32, 129.21, 129.01, 128.65, 128.56, 128.43, 128.03, 127.74, 127.15, 127.12, 115.29, 115.24, 114.17, 114.02, 113.89, 113.42, 113.40, 113.11, 112.68, 112.33 (C-a'), 64.78 (C-h'), 60.56 (C-j'), 56.51, 56.04, 55.95, 55.88, 55.85, 55.71, 55.58, 55.49, 55.20 (C-c'), 31.85, 30.36, 28.99, 28.71, 27.24 (C-b'), 10.77 (C-k').

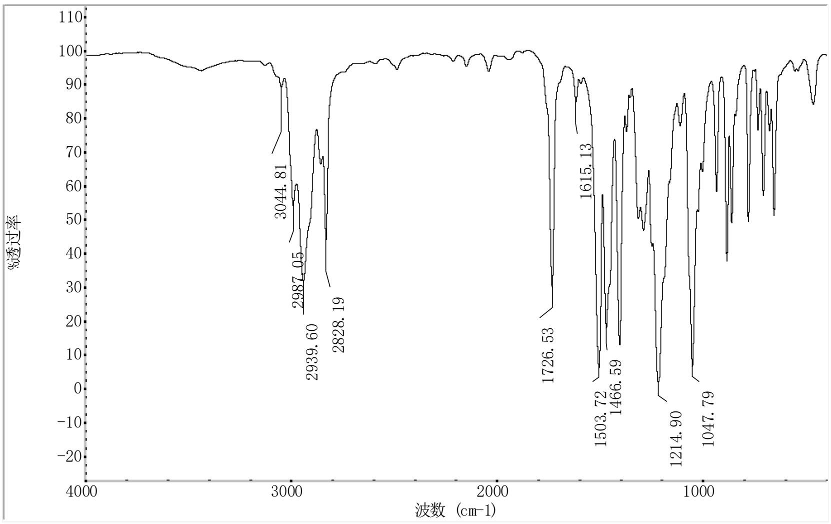

Figure S6. FTIR spectrum of P2.

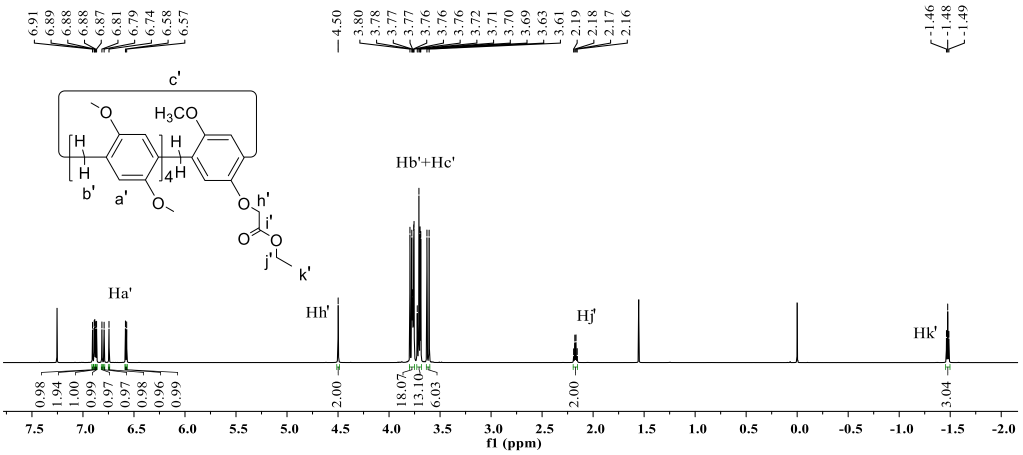

Figure S7. ^{1}H-NMR spectrum of P2 in $CDCl_3$.

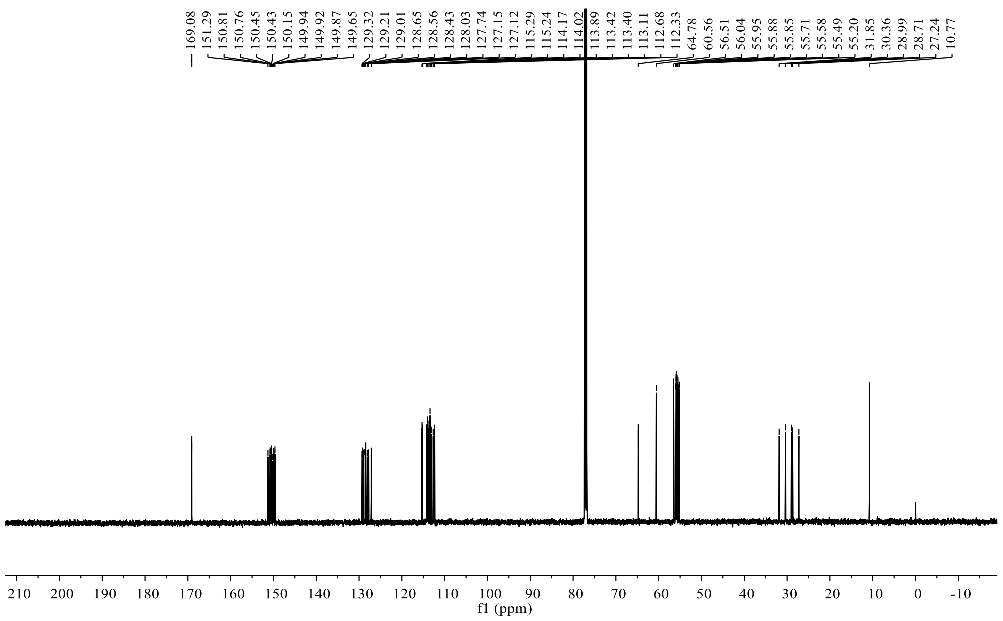

Figure S8. ^{13}C-NMR spectrum of P2 in $CDCl_3$.

S2. ^{1}H NMR spectra of P1 at variant concentrations

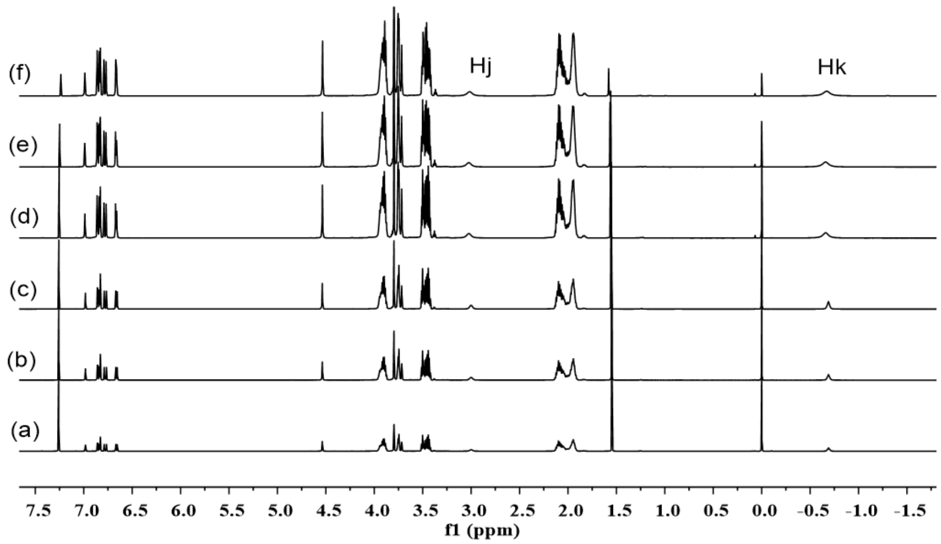

Figure S9 ^{1}H NMR spectra (600 MHz, 298 K) of P1 at variant concentrations in $CDCl_3$ indicate its concentration-independent property: (a) 3.72 mM, (b) 7.44 mM, (c) 11.16 mM, (d) 22.32 mM, (e) 44.64 mM, (f) 89.28 mM.

S3. ^{1}H NMR complexation analysis of P1 with G2-G5

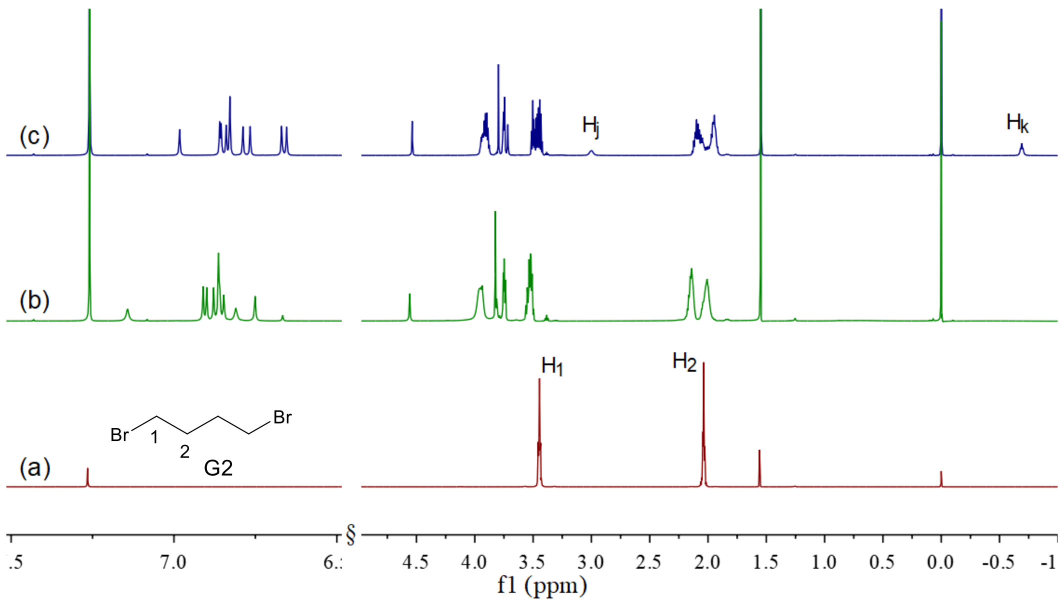

Figure S10 The ^{1}H NMR (600 MHz, $CDCl_3$, 298 k) spectra of (a) G2, (b). P1 + 1eq G2 (3.72 mM), (c) P1.

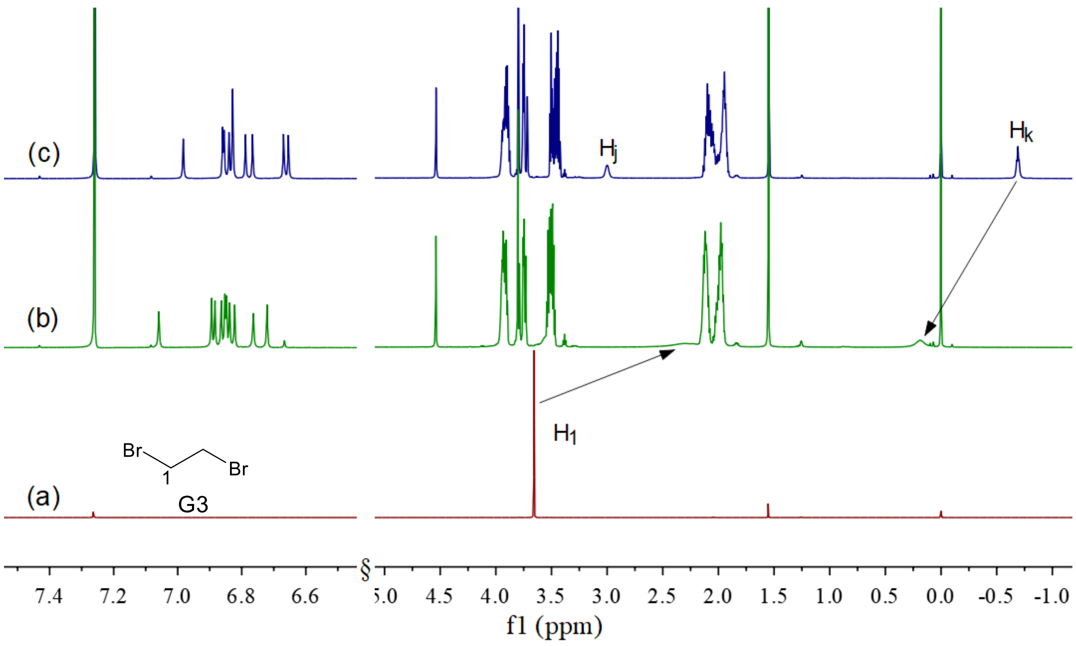

Figure S11 The ^{1}H NMR (600 MHz, $CDCl_3$, 298 k) spectra of (a) G3, (b). P1 + 1eq G3 (3.72 mM),

(c) P1.

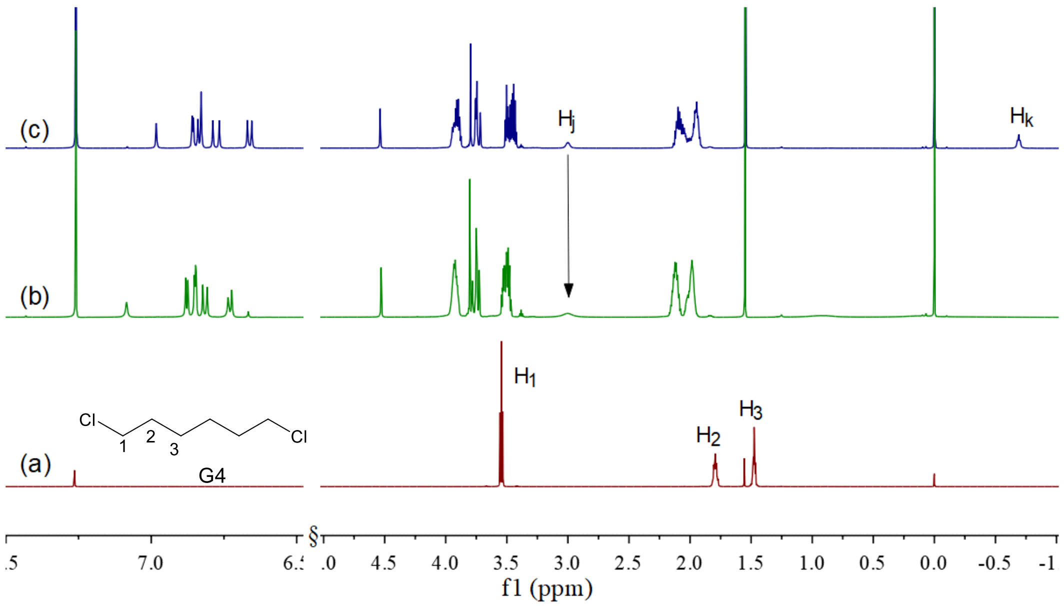

Figure S12 The ^{1}H NMR (600 MHz, $CDCl_3$, 298 k) spectra of (a) G4, (b). P1 + 1eq G4 (3.72 mM), (c) P1.

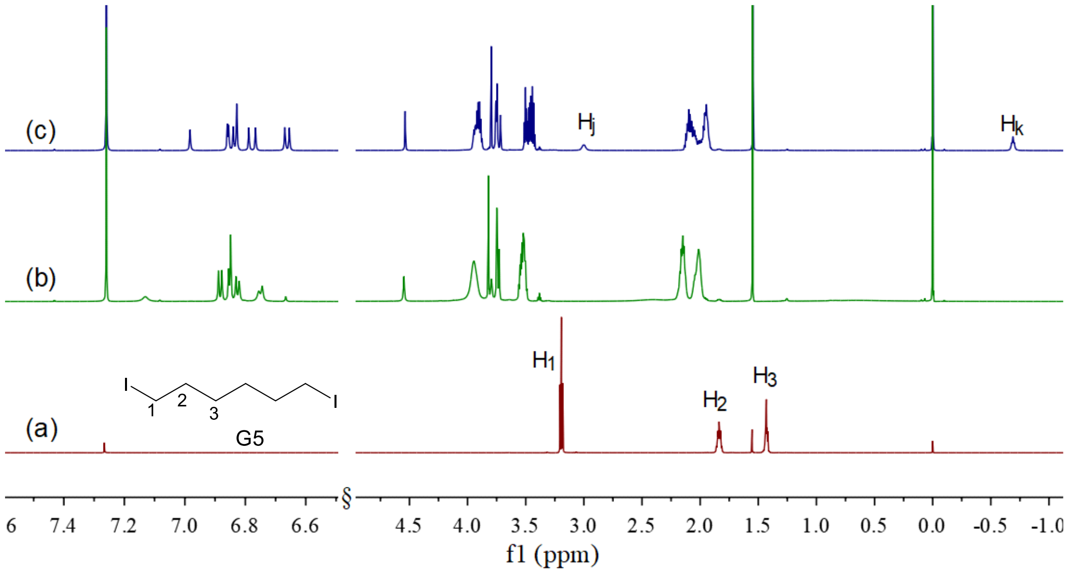

Figure S13 The ^{1}H NMR (600 MHz, $CDCl_3$, 298 k) spectra of (a) G5, (b). P1 + 1eq G5 (3.72 mM), (c) P1.

S4. UV-Vis absorption of complexation between P1 and G1

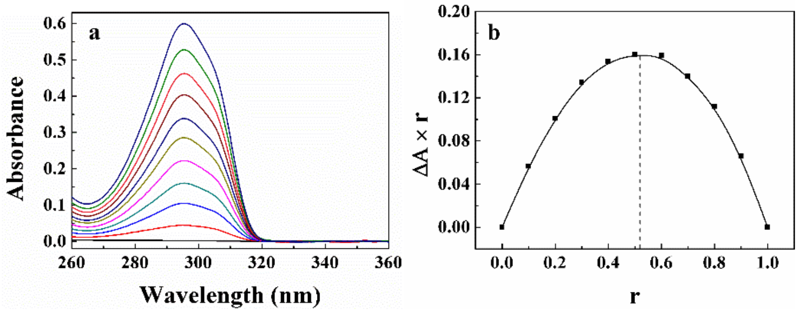

Figure S14. (a) UV spectra of the complex of P1 and G1 (curves from bottom to top, molar ratios from 0:10 to 10:0) in $CHCl_3$ solution (2×10^{-5} mM). (b) Job's plot of ΔA×r vs r detected by UV absorption at 295.5 nm. The stoichiometry of the complexation between P1 and G1 were detected with the continuous Job's variation method [S4].

S5. The 2D NOESY NMR spectrum of pseudo[1]rotaxane P1.

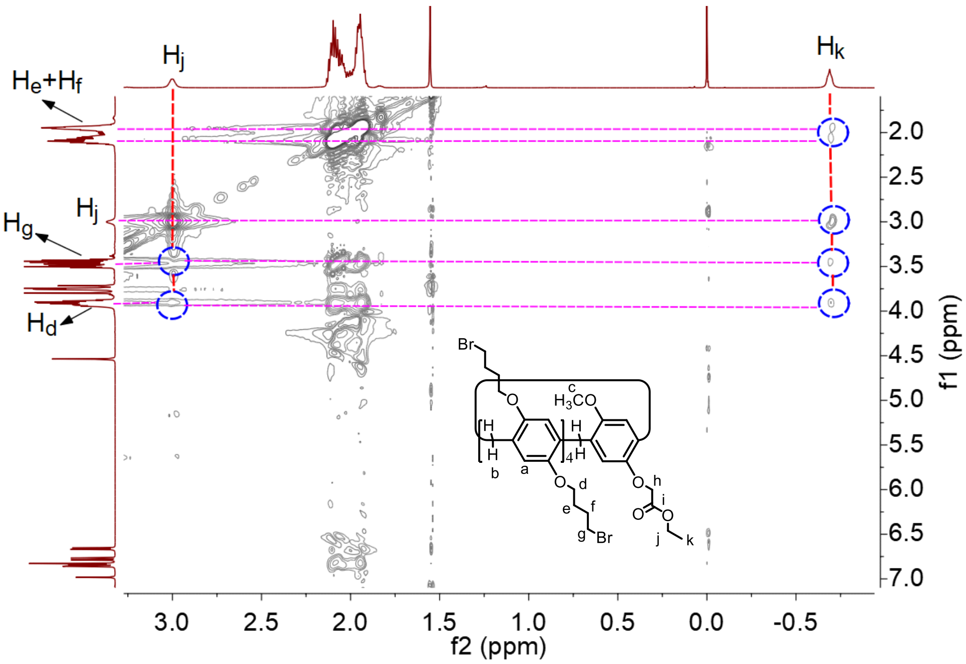

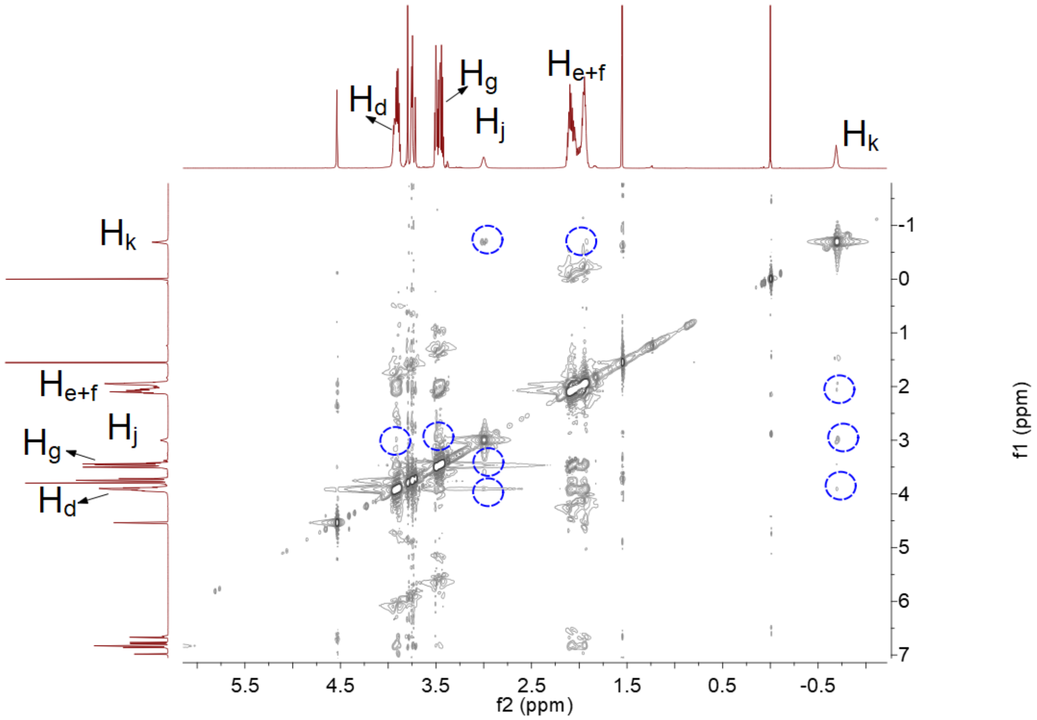

Figure S15 The 2D NOESY NMR (600 MHz, CDCl3, 298 k) spectrum of P1.

The strong NOE correlation of the proton H_j with the protons H_g and H_d, respectively. Meanwhile, the correlation between proton H_k and protons H_e, H_f, H_g, and H_d could be also observed, which demonstrated that the alkyl chain was bound by the cavity of pillar[5]arene and formed a self-inclusion complex.

S6. The ^{1}H NMR exchange experiments of P1 and P2.

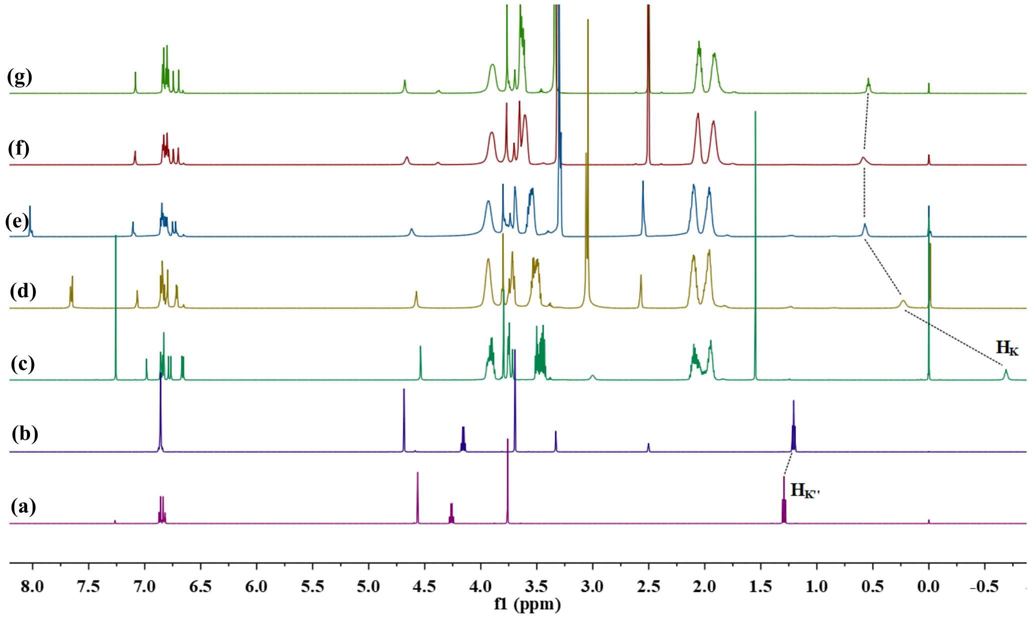

Figure S16 The ^{1}H NMR (600 MHz, 298 k) spectra of (a) M in $CDCl_3$, (b) M in DMSO-d6 (c) P1 in $CDCl_3$, (d) P1 in DMSO-d6/$CDCl_3$=0.25 (v/v), (e) P1 in DMSO-d6/$CDCl_3$=0.75 (v/v), (f) P1 in DMSO-d6/$CDCl_3$=0.9 (v/v), (g) P1 in DMSO-d6 (7.44 mM).

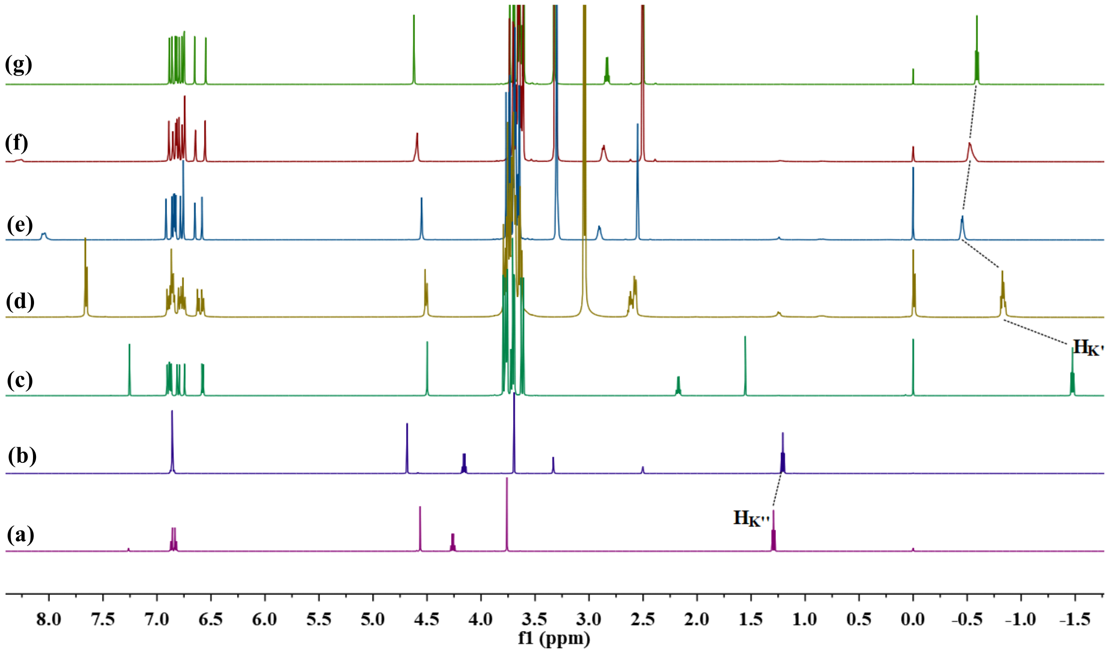

Figure S17 The ^{1}H NMR (600 MHz, 298 k) spectra of (a) M in $CDCl_3$, (b) M in DMSO-d6 (c) P2 in $CDCl_3$, (d) P2 in DMSO-d6/$CDCl_3$=0.25 (v/v), (e) P2 in DMSO-d6/$CDCl_3$=0.75 (v/v), (f) P2 in DMSO-d6/$CDCl_3$=0.9 (v/v), (g) P2 in DMSO-d6 (7.44 mM).

S7. The calculation of relative disassembly rate

Table S2. The chemical shifts of ^{1}H NMR (600 MHz, 298 k) exchange experiments of P1 and P2

	M	P1			P2		
Solvent	$\delta_{Hk''}$/ppm	δ_{Hk}/ppm	$\Delta\delta_{Hk}$ /ppm	$\Delta\delta_{Hk}/\Delta\delta_0$	$\delta_{Hk'}$/ppm	$\Delta\delta_{Hk'}$ /ppm	$\Delta\delta_{Hk'}/\Delta\delta_0$
$CDCl_3$	1.2926	-0.6893	0	0	-1.4752	0	0
DMSO/$CDCl_3$ =0.25 (v/v)		0.2275	0.9168	46.26%	-0.8311	0.6441	23.27%
DMSO/$CDCl_3$ =0.75 (v/v)		0.5716	1.2609	63.62%	-0.4532	1.022	36.92%
DMSO/$CDCl_3$		0.5869	1.276	64.39%	-0.5272	0.948	34.25%

=0.9 (v/v)		2				
DMSO	0.5392	1.228<br>5	61.99%	-0.5906	0.8846	31.96%

$\Delta\delta_0 = \delta_{Hk''(CDCl3)} – \delta_{Hk(CDCl3)} = 1.9819$; $\Delta\delta_0 = \delta_{Hk''(CDCl3)} – \delta_{Hk'(CDCl3)} = 2.7678$.

The disassembly rate (α) of S $\rightleftharpoons$ U in DMSO/$CDCl_3$ system relative to $CDCl_3$, was calculated according to the Eq. 1. Here, δ_S and δ are the chemical shifts of self-inclusion complex P1 (H_k) or P2 ($H_{k'}$) in chloroform and different ratio of DMSO/$CDCl_3$, respectively. The δ_U are the chemical shifts of uncomplex P1 (H_k) or P2 ($H_{k'}$), which is close to the chemical shift of the M ($H_{k''}$)

$$\delta = (1–\alpha)\times\delta_S + \alpha\times\delta_U \qquad (1)$$

$$\alpha\ (\%) = [\delta–\delta_S]/(\delta_U–\delta_S) = \Delta\delta/\Delta\delta_0$$

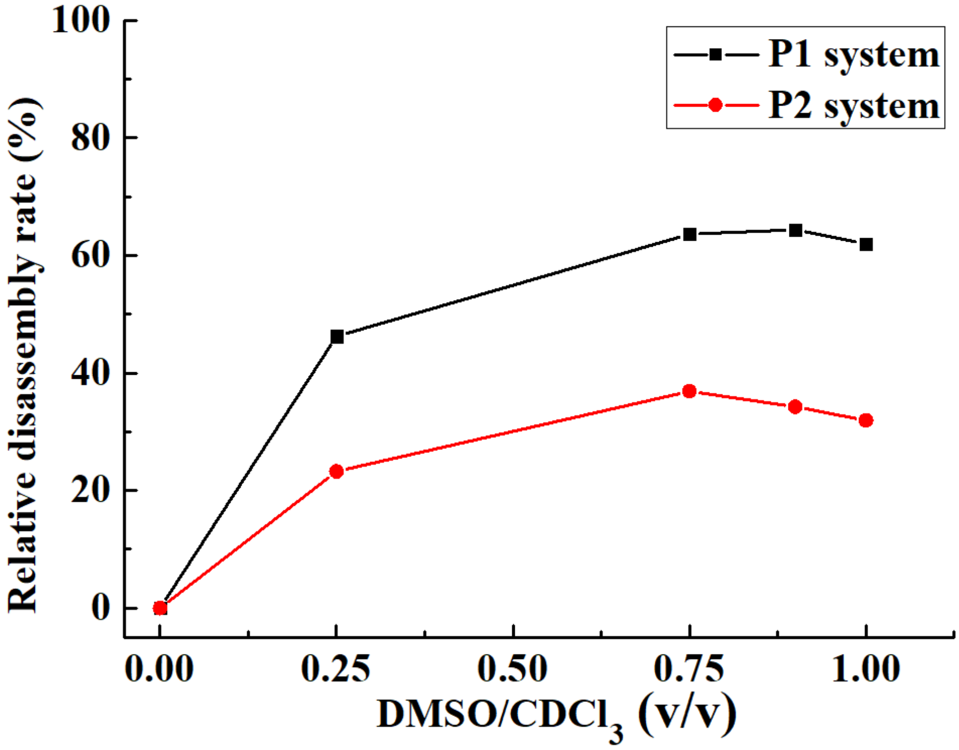

Figure S18 The relative disassembly rates of P1 and P2 systems from S structure to U structre in different ratio of DMSO-$CDCl_3$

S8. The ^{1}H NMR spectra of the complexation of P1 and P2 with G3

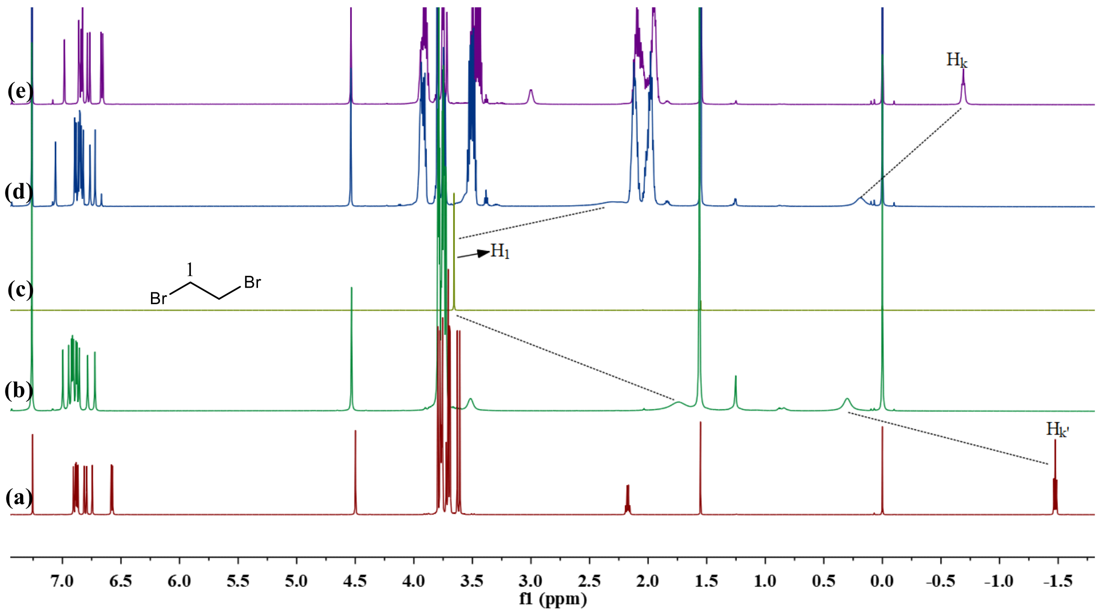

Figure S19 The ^{1}H NMR (600 MHz, 298 k) spectra of (a) P2 in $CDCl_3$,

(b) G3⊂P2, (c) G3, (d) G3⊂P1, (e) P1

Table S3. The chemical shifts of ^{1}H NMR spectra of the complexation of P1 and P2 with G3

	δ_{H1}/ppm	$\Delta\delta_{H1}$/ppm	$\delta_{H(CH3)}$/ppm	$\Delta\delta_{H(CH3)}$/ppm
G3	3.6572			
P1			-0.6893	
G3⊂P1	2.2957	1.3615	0.1828	-0.8721
P2			-1.4752	
G3⊂P2	1.7450	1.9122	0.3005	-1.7757

$\Delta\delta_{H1}= \delta_{H1(G3)}–\delta_{H1(\mathbf{G3\subset P1})}$=1.3615 ppm; $\Delta\delta_{H}= \delta_{H(P1)}–\delta_{H(\mathbf{G3\subset P1})}$=-0.8721 ppm;

$\Delta\delta_{H1}= \delta_{H1(G3)}–\delta_{H1(\mathbf{G3\subset P2})}$=1.9122 ppm; $\Delta\delta_{H}= \delta_{H(P2)}–\delta_{H(\mathbf{G3\subset P2})}$=-1.7757 ppm.

Table S4. Chemical shifts in different host–guest complexation ($CDCl_3$, 298 K).

Compound.	ΔH_j	ΔH_k	ΔH_1	ΔH_2	ΔH_3
P1 + G1	Disappeared	Disappeared	Disappeared	Disappeared	Disappeared
P1 + G2	Disappeared	Disappeared	Disappeared	Disappeared	–
P1 + G3	Disappeared	0.82	-1.37	–	–
P1 + G4	0.00	Disappeared	Disappeared	Disappeared	Disappeared

P1 + G5	Disappeared	Disappeared	Disappeared	Disappeared	Disappeared

$\Delta\delta H = H_{complex} - H_{free}$

S9. The process of Gibbs free energy

Density functional theory (DFT) calculations were carried out to optimize the structures of pseudo[1]rotaxane P1 and P2 at the B3LYP/6-31G(d, p) level in $CHCl_3$ by employing the Gaussian 09 program package. Frequency calculations have been carried out on the optimized geometries to confirm the Gibbs free energy (ΔG) according to eq. (2). The data of P2 as follows:

$$\Delta G = G_T - E_{ele} - E_{zpe} \qquad (2)$$

$$E = E_T - E_{ele} - E_{zpe}$$

	(Hartree)
Sum of electronic and zero-point Energies=	-2763.386297
Sum of electronic and thermal Energies=	-2763.327410
Sum of electronic and thermal Enthalpies=	-2763.326466
Sum of electronic and thermal Free Energies=	-2763.480368

ΔG= [-2763.480368 – (-2763.386297)]*2625.5=-246.983 KJ/moL

ΔE= [-2763.327410–(-2763.386297)]*2625.5=154.608 KJ/moL

The data of P1:

	(Hartree)
Sum of electronic and zero-point Energies=	-24275.244869
Sum of electronic and thermal Energies=	-24275.143419
Sum of electronic and thermal Enthalpies=	-24275.142474
Sum of electronic and thermal Free Energies=	-24275.417094

ΔG= [-24275.417094 – (-24275.244869)]*2625.5=-452.177 KJ/moL

ΔE= [-24275.143419–(-24275.244869)]*2625.5=266.357 KJ/moL

Where G_T, E_T, and $E_{ele} + E_{zpe}$ represent the Sum of electronic and thermal Free Energies, Sum of electronic and thermal Energies, and Sum of electronic and zero-point Energies, respectively (1

Hartree =2625.5 KJ/moL).

S10. The Optimized structures

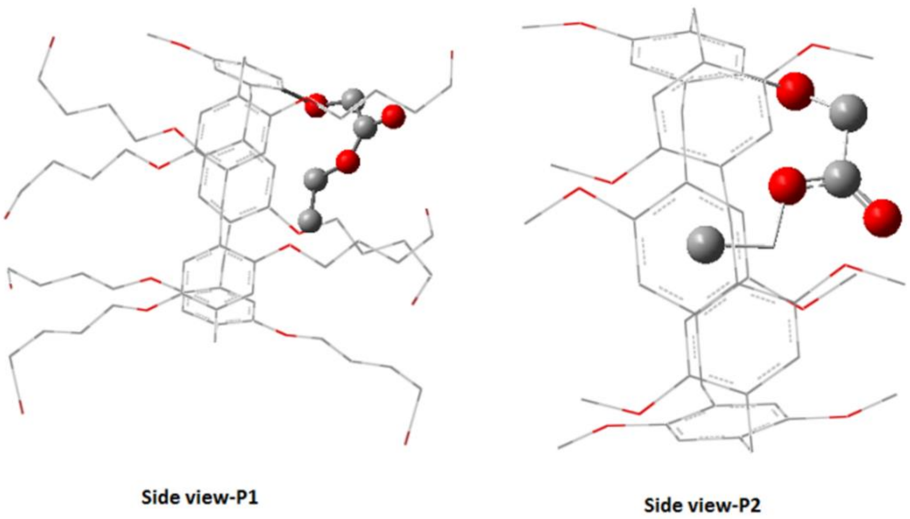

Figure 20. Optimized geometries of the self-inclusion structures of P1 and (b) P2 in $CHCl_3$. (Oxygen atoms are shown in red, bromine atoms are shown in red brown. Note that the H atoms are not presented in the figure, for convenience).

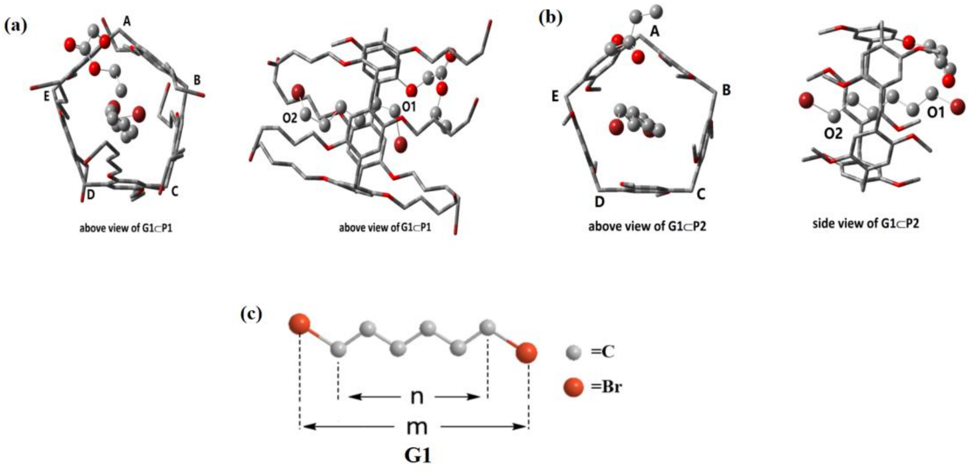

Figure S21 Optimized structures of (a) G1$\subset$P1,(b) G1$\subset$P2 and (c) free G1.

Table S5. The distances between the CH_2 group linking the Br in locked G1 (O1 and O2) and the para-methylene bridges (A, B, C, D, and E) of G1$\subset$P1 and G1$\subset$P2.

	G1$\subset$P1	G1$\subset$P2
Bond lengths (Å)		

O1–A	5.40	6.22
O1–B	6.05	7.10
O1–C	5.71	7.09
O1–D	4.40	5.62
O1–E	4.75	5.36
$\bar{d}_1$	5.26	6.28
O2–A	7.39	5.69
O2–B	6.44	4.64
O2–C	5.95	4.79
O2–D	6.41	5.73
O2–E	7.58	6.42
$\bar{d}_2$	6.75	5.45
$\Delta d=\bar{d}_2-\bar{d}_1$	1.49	-0.83

Table S6 The chain lengths of free G1, G1⊂P1 and G1⊂P2

	Free G1	G1⊂P1	G1⊂P2
Bond lengths (Å)			
m	9.70	7.94	9.63
n	6.40	6.39	6.31

S11. Coordinates of Optimized Structures P1, P2, G1⊂P1 and G1⊂P2

(1) Cartesian Coordinates of Optimized Structures P1

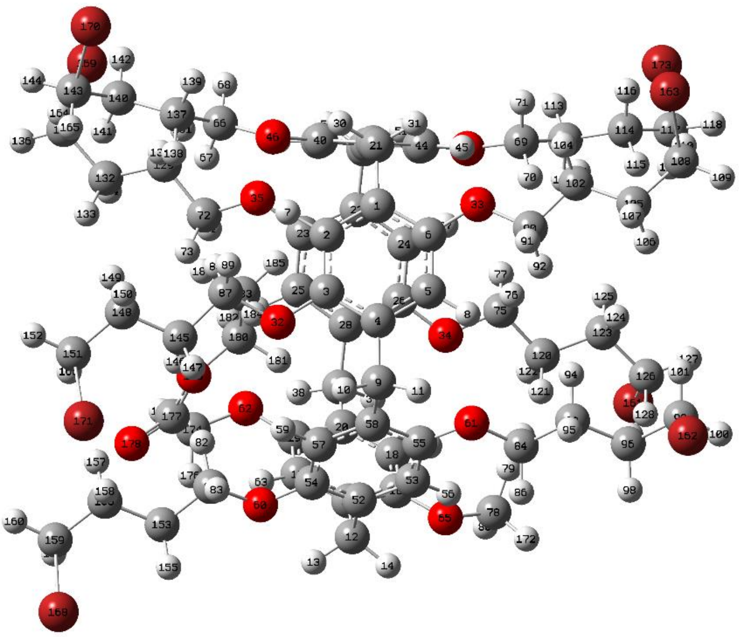

Center number	Atom	X	Y	Z
1	C	0.20715100	0.51789300	3.71276300
2	C	-1.12415000	0.21102200	3.41505400
3	C	-1.54325800	-1.10443500	3.20621500
4	C	-0.62475400	-2.16470100	3.29265600
5	C	0.69770300	-1.85901900	3.62502600
6	C	1.11933200	-0.54226600	3.83361600
7	H	-1.82270400	1.03407100	3.32528200
8	H	1.40244300	-2.67790000	3.69789800
9	C	-1.05376600	-3.60334900	3.04918200
10	H	-2.02776800	-3.76611300	3.51977600
11	H	-0.33556200	-4.26524000	3.54165900
12	C	-1.47847600	-5.00945100	-2.63971900

13	H	-2.49688400	-5.31098600	-2.89275000
14	H	-0.80981000	-5.83391500	-2.89525100
15	C	-1.09496000	-3.78027900	-3.44898800
16	C	0.19541700	-3.62026100	-3.98014700
17	C	-2.00482300	-2.73123000	-3.60830400
18	C	0.54953900	-2.43469600	-4.63112700
19	C	-1.64420300	-1.54387900	-4.24916800
20	C	-0.35083000	-1.37327600	-4.76673500
21	C	0.65294000	1.96679800	3.83318600
22	C	1.30007800	3.00066200	-2.61117700
23	C	0.01471700	3.01230700	-3.17637500
24	C	2.18946700	2.00303700	-3.02150300
25	C	-0.35897900	2.02323900	-4.09193000
26	C	1.82094000	1.02508500	-3.94936500
27	H	3.17796400	2.00116100	-2.57955800
28	C	0.51998700	1.00771500	-4.48021400
29	H	-1.35561900	2.01523200	-4.51549500
30	H	-0.12942600	2.54841800	4.32602300
31	H	1.55188900	2.01694600	4.45146600
32	O	-2.84000300	-1.44157900	2.87764200
33	O	2.42315900	-0.21013000	4.14526700

34	O	2.68182700	0.04746600	-4.39899300
35	O	-0.82893000	4.02749300	-2.77342700
36	C	0.07340400	-0.07903800	-5.44703000
37	H	0.89283900	-0.30297400	-6.13581300
38	H	-0.76242300	0.30349800	-6.03944000
39	C	0.23308200	3.75928300	0.47123100
40	C	-0.03524700	3.28683700	1.75960900
41	C	1.47092100	3.54783200	-0.14450400
42	C	0.94514500	2.57372200	2.46919200
43	C	2.45977400	2.85908900	0.57664600
44	C	2.18944300	2.38163000	1.86221100
45	H	2.93548800	1.82270100	2.41421400
46	O	-1.24442500	3.47044500	2.39755500
47	O	3.67361400	2.68331700	-0.05601500
48	H	-0.52567200	4.28757000	-0.09317600
49	C	1.71698300	4.02573000	-1.56750000
50	H	1.15983300	4.95056700	-1.73456600
51	H	2.77972200	4.24742900	-1.69131300
52	C	-1.39290700	-4.72103700	-1.14821400
53	C	-0.15276000	-4.72762700	-0.50230400
54	C	-2.53199500	-4.36576700	-0.40803300

55	C	-0.02676000	-4.36342000	0.84022100
56	H	0.71588600	-5.00514500	-1.08744500
57	C	-2.40307400	-4.01128600	0.93981700
58	C	-1.16217600	-3.99614600	1.58226700
59	H	-3.26977800	-3.71464000	1.51519500
60	O	-3.73113100	-4.39475600	-1.08046400
61	O	1.17673200	-4.33761900	1.51246500
62	O	-2.50427900	-0.46321300	-4.37683100
63	H	-2.99549100	-2.86331700	-3.18764600
64	H	1.54879600	-2.29760500	-5.02366900
65	O	1.06242100	-4.67551300	-3.80317600
66	C	-2.17237700	4.39556000	1.84486600
67	H	-2.55732300	4.02306800	0.88317600
68	H	-1.67673100	5.35836700	1.65345700
69	C	4.77415300	2.21852600	0.71494900
70	H	4.58798500	1.19384300	1.07069200
71	H	4.90868800	2.85632400	1.60083500
72	C	-1.99483400	4.28658300	-3.54633500
73	H	-2.71442400	3.45904000	-3.44778700
74	H	-1.72995200	4.36806100	-4.61054400
75	C	4.06906700	0.19167900	-4.11824100

76	H	4.25511400	0.07774200	-3.03928700
77	H	4.40851100	1.19673400	-4.40803500
78	C	2.31910100	-4.62529100	-4.45366500
79	H	2.94923000	-3.81498000	-4.06237400
80	H	2.21070700	-4.49551500	-5.53791100
81	C	-4.90246300	-3.90349100	-0.42682200
82	H	-4.78045900	-2.82950700	-0.22834100
83	H	-5.04887000	-4.41512900	0.53387700
84	C	2.36348400	-4.62432700	0.78844500
85	H	2.40752300	-4.01839000	-0.12804700
86	H	2.38183100	-5.68324000	0.48676700
87	C	-3.89456500	-0.54316500	3.21104400
88	H	-3.76223900	0.42066200	2.70048300
89	H	-3.89267900	-0.35092300	4.29432100
90	C	3.24978200	-1.21404000	4.72791500
91	H	2.71236400	-1.70757200	5.55035200
92	H	3.50025000	-1.98837200	3.98828300
93	C	3.54384300	-4.29299400	1.69184600
94	H	3.51471100	-3.21919800	1.91540400
95	H	3.43379100	-4.82060400	2.64438100
96	C	4.88909500	-4.65749700	1.05596800

97	H	4.94877600	-4.23558600	0.04082600
98	H	4.97722000	-5.74462800	0.94768800
99	C	6.09796000	-4.12352500	1.80784400
100	H	7.03889600	-4.41839400	1.34490500
101	H	6.06703700	-3.03958900	1.92366900
102	C	4.51779900	-0.54259000	5.23715100
103	H	5.03541000	-0.07079100	4.39129100
104	H	4.24771300	0.25995700	5.93048700
105	C	5.45213200	-1.53714600	5.93453400
106	H	5.70125400	-2.36438400	5.25378100
107	H	4.94473200	-1.98727700	6.79609900
108	C	6.77036600	-0.93588700	6.39421100
109	H	7.41900800	-1.67507600	6.86225500
110	H	7.30684000	-0.43520300	5.58741100
111	C	6.01313600	2.25991400	-0.16973300
112	H	5.86310600	1.58999300	-1.02651500
113	H	6.13310800	3.27011300	-0.57304800
114	C	7.27963100	1.85625400	0.59240900
115	H	7.14861400	0.85447300	1.02983900
116	H	7.45090400	2.54221800	1.43000700
117	C	8.53323100	1.79399900	-0.26695900

118	H	9.40672300	1.47705400	0.30147300
119	H	8.40872900	1.15010500	-1.13853000
120	C	4.81810100	-0.87336100	-4.90797000
121	H	4.48302000	-1.86562700	-4.57922800
122	H	4.55592800	-0.78269700	-5.96653000
123	C	6.33694300	-0.75608200	-4.74547400
124	H	6.60548700	-0.82640900	-3.68005600
125	H	6.67893300	0.22680900	-5.08965300
126	C	7.12688200	-1.83907400	-5.46501400
127	H	8.20002300	-1.73828700	-5.30794900
128	H	6.80753700	-2.84378000	-5.18598600
129	C	-2.60958400	5.58604800	-3.04391800
130	H	-2.85825300	5.47459000	-1.98064900
131	H	-1.86585300	6.38567800	-3.11510300
132	C	-3.86286800	5.97545500	-3.83467500
133	H	-4.61170900	5.17174900	-3.76724000
134	H	-3.62000600	6.08813300	-4.89776100
135	C	-4.54114100	7.24513100	-3.34290100
136	H	-5.44586000	7.47239000	-3.90529900
137	C	-3.30616700	4.56561700	2.84726100
138	H	-3.76085900	3.58513000	3.03875900

139	H	-2.89365600	4.91624300	3.79830200
140	C	-4.37442300	5.54785100	2.35623000
141	H	-4.80786300	5.18495600	1.41169700
142	H	-3.92345400	6.52343500	2.13977800
143	C	-5.53345300	5.74030000	3.32278900
144	H	-6.29779000	6.40482700	2.92199300
145	C	-5.19399900	-1.19453200	2.75719900
146	H	-5.16632500	-1.29736100	1.66554500
147	H	-5.25394200	-2.20555700	3.17143100
148	C	-6.43639200	-0.40076800	3.17166900
149	H	-6.33794300	0.64703100	2.84832600
150	H	-6.53102000	-0.38446000	4.26371000
151	C	-7.72897300	-0.91660900	2.55697800
152	H	-8.59946200	-0.35253100	2.88949800
153	C	-6.09240600	-4.15749900	-1.34177900
154	H	-5.83972500	-3.81384900	-2.35024900
155	H	-6.29048000	-5.23316800	-1.39535100
156	C	-7.34142700	-3.41064200	-0.86186800
157	H	-7.15432500	-2.33248300	-0.96621300
158	H	-7.54520300	-3.60864200	0.19734200
159	C	-8.58674500	-3.70503700	-1.67983900

160	H	-9.44445300	-3.11786400	-1.35440800
161	Br	6.89166200	-1.75834000	-7.43392600
162	Br	6.20615600	-4.82973600	3.66215700
163	Br	6.51716000	0.46654900	7.77948300
164	H	-4.77035600	7.21088400	-2.27724500
165	H	-5.98836200	4.79558300	3.62256000
166	H	-7.69139200	-0.93005500	1.46736100
167	H	-8.42554800	-3.56356500	-2.74858600
168	Br	-9.18563600	-5.59622800	-1.50127400
169	Br	-3.38870200	8.84613900	-3.56587900
170	Br	-4.95923500	6.59315800	5.02041500
171	Br	-8.11416500	-2.79573800	3.07210200
172	H	2.80283400	-5.58304600	-4.25306900
173	Br	9.02576000	3.56683500	-1.01023800
174	C	-3.89321800	-0.68083300	-4.25157700
175	H	-4.37120300	0.17705100	-4.73719300
176	H	-4.20876400	-1.58077000	-4.79016100
177	C	-4.54668000	-0.78967200	-2.85983000
178	O	-5.67820000	-1.23095400	-2.81859300
179	O	-3.96689300	-0.37302600	-1.72809800
180	C	-2.65378400	0.25172900	-1.62757800

181	H	-1.88726200	-0.48699500	-1.86626400
182	H	-2.58194700	1.07197100	-2.34397500
183	C	-2.51586100	0.73757500	-0.19988600
184	H	-2.56864200	-0.09951300	0.50055900
185	H	-1.54865600	1.23176100	-0.07286300
186	H	-3.30688900	1.45418800	0.04457000

(2) Cartesian Coordinates of Optimized Structures P2

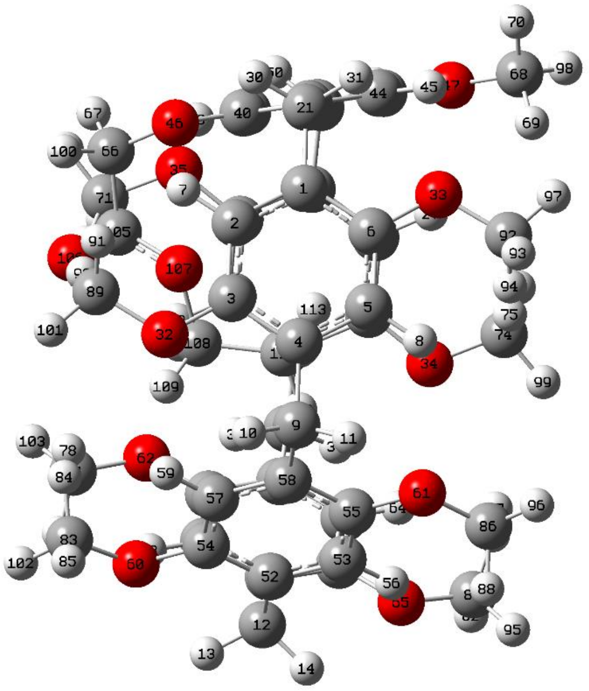

1	C	0.83159800	-4.15107000	0.23254800
2	C	1.61218500	-3.94458000	-0.90876300
3	C	2.83292100	-3.26314500	-0.85193200
4	C	3.30352600	-2.76329900	0.37364300
5	C	2.53506900	-2.98865100	1.52016000
6	C	1.31913500	-3.67680600	1.46243100
7	H	1.23161100	-4.31457700	-1.85246900
8	H	2.90681900	-2.59566600	2.45892000
9	C	4.59122400	-1.95662400	0.45084100
10	H	5.25768700	-2.26851700	-0.35667300
11	H	5.09171100	-2.16602100	1.39868600
12	C	3.31590100	3.76147300	-0.00281400
13	H	3.70771000	4.17414200	-0.93565400
14	H	3.76959400	4.31396100	0.82401400
15	C	1.80754100	3.96092000	0.03098300
16	C	1.11757600	4.10531600	1.24617300
17	C	1.06469200	3.97019800	-1.15400500
18	C	-0.27532600	4.22737100	1.25502500
19	C	-0.32763900	4.09898900	-1.14505900
20	C	-1.01990300	4.21597800	0.07229100
21	C	-0.50940100	-4.86395500	0.14110400

22	C	-4.40672200	0.36581200	0.06674100
23	C	-4.17360900	1.06755600	-1.12676500
24	C	-4.05151400	0.97898300	1.27279200
25	C	-3.58810200	2.33633400	-1.09502400
26	C	-3.46370100	2.24750600	1.30462900
27	H	-4.22955200	0.42664900	2.18806000
28	C	-3.21575100	2.94203100	0.10816400
29	H	-3.38061200	2.87395100	-2.01141300
30	H	-0.51186400	-5.48649900	-0.75791700
31	H	-0.61945100	-5.52973100	1.00197100
32	O	3.63550200	-3.03919700	-1.94733200
33	O	0.54025400	-3.94137000	2.56720500
34	O	-3.10076000	2.89034700	2.46821500
35	O	-4.54333300	0.43248300	-2.29399600
36	C	-2.53811600	4.30409900	0.11063700
37	H	-2.83707500	4.85168800	1.00809300
38	H	-2.88536000	4.87152400	-0.75691800
39	C	-3.16481000	-2.38974800	-1.08406300
40	C	-2.08276800	-3.26873600	-1.07577200
41	C	-3.87816100	-2.09776900	0.07846500
42	C	-1.70445100	-3.92088700	0.10464100

43	C	-3.50475400	-2.75075700	1.26609700
44	C	-2.44395000	-3.65978200	1.26471800
45	H	-2.13838600	-4.15437500	2.17785800
46	O	-1.34717600	-3.57637400	-2.21160600
47	O	-4.22845500	-2.42575200	2.39422200
48	H	-3.44924500	-1.86805100	-1.99024600
49	C	-4.97647100	-1.04480200	0.05797100
50	H	-5.58843900	-1.17893600	-0.83737000
51	H	-5.62248700	-1.17972400	0.92777500
52	C	3.71389100	2.29676000	0.10359600
53	C	3.92213400	1.70668500	1.35395800
54	C	3.83877500	1.49099600	-1.04180500
55	C	4.22056800	0.34676900	1.48201100
56	H	3.80933400	2.33429300	2.22897500
57	C	4.15475300	0.13458600	-0.91329200
58	C	4.33599800	-0.46089300	0.33950900
59	H	4.23420100	-0.50092400	-1.78685400
60	O	3.62690600	2.11121500	-2.25129500
61	O	4.40565300	-0.27997200	2.69675700
62	O	-1.09665500	4.12035600	-2.28815800
63	H	1.60504200	3.85042000	-2.08465000

64	H	-0.81818600	4.30932300	2.18824100
65	O	1.88885200	4.11798300	2.38883700
66	C	-1.17813900	-2.60846200	-3.23306000
67	H	-2.13307100	-2.17371500	-3.55757000
68	C	-3.93260300	-3.11289900	3.59577500
69	H	-2.90802600	-2.91544800	3.93850600
70	H	-4.06478300	-4.19741300	3.48733300
71	C	-4.41209100	1.14423700	-3.51157000
72	H	-3.36390800	1.38292000	-3.73496700
73	H	-4.99225200	2.07605100	-3.50187400
74	C	-3.38248000	2.24965000	3.69913300
75	H	-2.84419400	1.29779400	3.79671800
76	H	-4.45700500	2.06271000	3.82449600
77	C	-0.43359800	4.08124500	-3.54019400
78	H	0.11391000	3.14070800	-3.68435700
79	H	0.26506500	4.91985800	-3.65584800
80	C	1.24214100	4.34640800	3.62755200
81	H	0.52944500	3.54721100	3.87060100
82	H	0.71211100	5.30770300	3.63897300
83	C	3.65855800	1.31515700	-3.42922100
84	H	2.87947500	0.54326000	-3.42090200

85	H	4.63904500	0.84288600	-3.57145000
86	C	4.37826100	0.51502900	3.86751900
87	H	3.39697000	0.98344600	4.02136400
88	H	5.14431400	1.30116500	3.84403000
89	C	3.22313900	-3.55158600	-3.20574300
90	H	2.30410500	-3.06800600	-3.55615800
91	H	3.07965300	-4.63933900	-3.17237700
92	C	1.01637700	-3.52420200	3.83311800
93	H	1.98587400	-3.97961000	4.07437900
94	H	1.11401200	-2.43224900	3.89393100
95	H	2.03038100	4.36457200	4.38269900
96	H	4.58607900	-0.16156000	4.69876400
97	H	0.27469800	-3.85770900	4.56170100
98	H	-4.63773200	-2.73951700	4.34095700
99	H	-3.04587800	2.93322200	4.48101700
100	H	-0.74755700	-3.13781000	-4.08415600
101	H	4.03300000	-3.32259400	-3.90103500
102	H	3.47533100	2.00228100	-4.25752100
103	H	-1.21447600	4.15958800	-4.29898100
104	H	-4.80347000	0.48698400	-4.29036000
105	C	-0.21705400	-1.47021400	-2.87284500

106	O	0.63067300	-1.05484700	-3.64344300
107	O	-0.45186000	-1.00127400	-1.65346500
108	C	0.41911300	0.04163200	-1.13396900
109	H	1.45407500	-0.27990200	-1.26031700
110	H	0.26070700	0.94896300	-1.72392900
111	C	0.05873800	0.23951400	0.32319100
112	H	0.69467300	1.02171500	0.74607600
113	H	-0.98604100	0.54174300	0.43046800
114	H	0.21941800	-0.68581200	0.88272800

(3) Cartesian Coordinates of Optimized Structures G1⊂P1

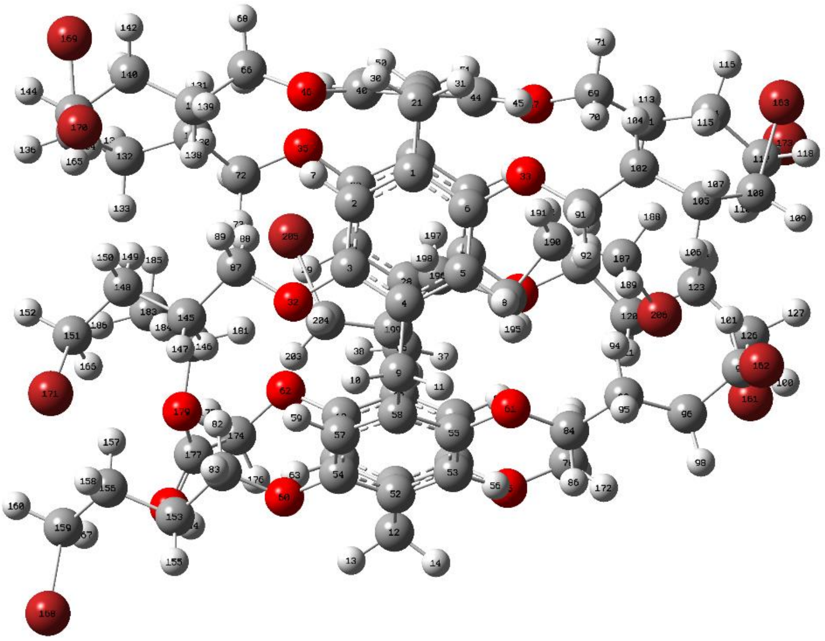

1	C	-0.04515800	-3.20911800	2.49226100
2	C	1.24318400	-2.68843400	2.53698700
3	C	1.49995900	-1.47438100	3.15826100
4	C	0.46408900	-0.77662300	3.78217500
5	C	-0.83112200	-1.27329800	3.69470000
6	C	-1.09334100	-2.46961600	3.03519700
7	H	2.01748100	-3.24256300	2.02796000
8	H	-1.61858200	-0.66981600	4.12182700
9	C	0.76377600	0.49964600	4.53666300
10	H	1.73609000	0.40021000	5.02418900
11	H	-0.00004000	0.65113400	5.30320200
12	C	0.81918700	5.12623100	1.01324200
13	H	1.77908400	5.64030100	1.06137400
14	H	0.01533100	5.82614500	1.24589000
15	C	0.60073100	4.54938700	-0.36854000
16	C	-0.70723800	4.34346800	-0.82741400
17	C	1.66529500	4.12329600	-1.15049300
18	C	-0.92331700	3.75762400	-2.06495900
19	C	1.44305900	3.56046500	-2.40446400
20	C	0.14801300	3.39248400	-2.87853600
21	C	-0.31605000	-4.51462000	1.77632000

22	C	-0.67927200	-1.48148300	-3.86205700
23	C	0.60484500	-0.96308900	-4.04186200
24	C	-1.75352700	-0.60944100	-3.72515200
25	C	0.77674700	0.41247900	-4.15718500
26	C	-1.57758700	0.76561000	-3.82471700
27	H	-2.71987500	-1.04617700	-3.51754900
28	C	-0.30519500	1.27939100	-4.07634300
29	H	1.75284900	0.85561700	-4.29417900
30	H	0.52618800	-5.19427200	1.91555200
31	H	-1.22356500	-4.96790500	2.17808200
32	O	2.75227100	-0.87149900	3.18292300
33	O	-2.38209100	-2.98043500	2.83934200
34	O	-2.61027800	1.68665600	-3.70738400
35	O	1.65129300	-1.87951600	-4.10464600
36	C	-0.11000200	2.76998700	-4.22992900
37	H	-1.01074000	3.20491700	-4.66752300
38	H	0.74042000	2.96351100	-4.88556200
39	C	0.46393100	-3.80175400	-1.85953300
40	C	0.62610700	-4.17044900	-0.53220700
41	C	-0.78205800	-3.44206600	-2.35910300
42	C	-0.48805700	-4.21370300	0.30602300

43	C	-1.88993600	-3.44725400	-1.50954200
44	C	-1.73444700	-3.85674700	-0.19147700
45	H	-2.55607800	-3.85962800	0.50907200
46	O	1.84913200	-4.45890900	0.06300300
47	O	-3.09942200	-2.97915200	-2.03787400
48	H	1.30359900	-3.70429800	-2.53172000
49	C	-0.90803600	-2.97471400	-3.78958200
50	H	-0.16680900	-3.48374900	-4.40911800
51	H	-1.90770300	-3.20757800	-4.16188400
52	C	0.81091300	3.97301800	1.99107700
53	C	-0.38562600	3.47697000	2.48685800
54	C	2.00675800	3.31695300	2.28706100
55	C	-0.40960300	2.34693700	3.29346500
56	H	-1.29131600	3.96788000	2.16609100
57	C	1.98389500	2.19000000	3.10266500
58	C	0.78500100	1.70500000	3.62332300
59	H	2.88560000	1.63845100	3.32816600
60	O	3.13866100	3.87333200	1.71801800
61	O	-1.59455400	1.79285600	3.78386000
62	O	2.49875900	3.12849000	-3.23960200
63	H	2.66112800	4.20211700	-0.73526200

64	H	-1.91667600	3.53793800	-2.42903800
65	O	-1.72637800	4.74716800	0.01985000
66	C	2.99823400	-4.81984300	-0.74868500
67	H	2.99542700	-4.29699500	-1.70798800
68	H	2.98378300	-5.89971400	-0.93886600
69	C	-4.32405100	-3.31077700	-1.32722000
70	H	-4.20572900	-3.13244700	-0.25385300
71	H	-4.57011800	-4.36895700	-1.47587800
72	C	2.98626800	-1.36178300	-4.33058300
73	H	3.17500300	-0.50019600	-3.68201000
74	H	3.10150800	-1.05354000	-5.37777500
75	C	-3.95989600	1.16277600	-3.59405100
76	H	-4.04340800	0.51406300	-2.71407800
77	H	-4.21356500	0.57850600	-4.48801400
78	C	-3.08737000	4.45672800	-0.38956800
79	H	-3.24392400	3.37956000	-0.50810200
80	H	-3.34500200	4.96703200	-1.32439800
81	C	4.40917500	3.16483700	1.72504800
82	H	4.35721900	2.33372100	1.01210400
83	H	4.64951100	2.77485400	2.72013500
84	C	-2.82150700	2.50881800	3.47564000

85	H	-2.94686400	2.58544800	2.38793300
86	H	-2.79030000	3.51981900	3.90059000
87	C	3.84051600	-1.53760300	2.47504600
88	H	3.50647200	-1.82586600	1.47436600
89	H	4.16362000	-2.43157300	3.02182300
90	C	-3.45639100	-2.39595100	3.61589500
91	H	-3.31924200	-2.61057200	4.68287200
92	H	-3.47142100	-1.30847900	3.48523100
93	C	-3.97327000	1.70326900	4.06292300
94	H	-3.91137200	0.68941000	3.65565700
95	H	-3.86899200	1.63076600	5.14793600
96	C	-5.31334700	2.35155100	3.67568900
97	H	-5.27898300	2.62934100	2.61304100
98	H	-5.46830000	3.26535900	4.25783900
99	C	-6.51521000	1.43160400	3.82213800
100	H	-7.44064700	1.93333100	3.54449700
101	H	-6.39362700	0.52854000	3.22379800
102	C	-4.76182400	-2.97381500	3.08270200
103	H	-4.82155700	-2.73675100	2.01576100
104	H	-4.75494100	-4.06192500	3.17444600
105	C	-5.95154500	-2.35495800	3.84328900

106	H	-5.66627700	-1.37119300	4.24094200
107	H	-6.20991800	-2.97104300	4.70934500
108	C	-7.18911700	-2.12744900	2.98968400
109	H	-7.98716800	-1.66042500	3.56534600
110	H	-6.96297100	-1.52516100	2.10839400
111	C	-5.42349200	-2.39332900	-1.85086300
112	H	-5.10908500	-1.35506400	-1.69462900
113	H	-5.56200800	-2.54066000	-2.92453300
114	C	-6.72970900	-2.66975500	-1.08789200
115	H	-6.50919900	-2.82950000	-0.02251100
116	H	-7.19018000	-3.59536100	-1.44676000
117	C	-7.73500800	-1.53066900	-1.15156600
118	H	-8.61497800	-1.74720100	-0.54730000
119	H	-7.28407000	-0.58532100	-0.84589000
120	C	-4.89001600	2.35642500	-3.43694700
121	H	-4.66819200	2.84375300	-2.48137500
122	H	-4.70144600	3.07530700	-4.23757700
123	C	-6.35030600	1.88168600	-3.46666200
124	H	-6.45145400	0.96834100	-2.86656200
125	H	-6.63756400	1.62334800	-4.49078500
126	C	-7.33546400	2.88521200	-2.89030100

127	H	-8.35657800	2.51023100	-2.94111500
128	H	-7.08246100	3.14221300	-1.86132100
129	C	3.96541500	-2.46957900	-3.96232200
130	H	3.88182700	-2.64641600	-2.88320700
131	H	3.69266700	-3.39184300	-4.48082300
132	C	5.40153700	-2.05387500	-4.31856800
133	H	5.57251500	-1.01824100	-3.99248200
134	H	5.54061800	-2.08212200	-5.40396200
135	C	6.46278500	-2.90617300	-3.63847200
136	H	7.46988500	-2.56840400	-3.87719600
137	C	4.23174700	-4.41063000	0.05112100
138	H	4.32302800	-3.31904700	0.01230000
139	H	4.08268700	-4.70011000	1.09466400
140	C	5.49833500	-5.07463700	-0.50052100
141	H	5.56489800	-4.91581200	-1.58521900
142	H	5.44843500	-6.15666800	-0.33916700
143	C	6.77848300	-4.52484400	0.11136300
144	H	7.66301800	-5.00185600	-0.30769500
145	C	4.95782300	-0.51036900	2.36799300
146	H	4.57974400	0.34581600	1.79957400
147	H	5.22828000	-0.16158700	3.36804400

148	C	6.18352500	-1.10274900	1.66324900
149	H	5.88369200	-1.44049700	0.66014200
150	H	6.54685900	-1.97895600	2.20930400
151	C	7.31367400	-0.09914400	1.47789800
152	H	8.16627500	-0.54399000	0.96650600
153	C	5.43920200	4.19114100	1.27043700
154	H	5.04255400	4.70618900	0.39336900
155	H	5.59225000	4.93066200	2.06043700
156	C	6.76745800	3.53209100	0.88199600
157	H	6.54475900	2.76987200	0.12442400
158	H	7.24414600	3.05592300	1.74507700
159	C	7.71673600	4.51307300	0.20972700
160	H	8.63885900	4.03019400	-0.11032500
161	Br	-7.33224900	4.60285900	-3.89529800
162	Br	-6.77934300	0.81065600	5.69321300
163	Br	-7.93670100	-3.83179500	2.29082000
164	H	6.32030900	-2.92429900	-2.55679000
165	H	6.84482900	-3.44295200	-0.00637900
166	H	6.98045500	0.78878000	0.94382600
167	H	7.21379000	4.99040900	-0.63013700
168	Br	8.29046900	5.97121600	1.43827700

169	Br	6.37911900	-4.80251100	-4.23207600
170	Br	6.86684000	-4.85323900	2.07276600
171	Br	8.02006300	0.56968600	3.21078800
172	H	-3.71548600	4.83152900	0.41863200
173	Br	-8.40698600	-1.23503400	-3.00058700
174	C	3.59795200	4.05461800	-3.39698300
175	H	4.08372900	3.78024700	-4.33880100
176	H	3.25122500	5.08924100	-3.46110700
177	C	4.63051500	4.01181800	-2.27530200
178	O	5.25542100	5.01193400	-1.95328900
179	O	4.85783600	2.83223000	-1.63462600
180	C	4.49329600	1.52589900	-2.22291800
181	H	3.44416100	1.31669300	-2.03632200
182	H	4.66502800	1.54356200	-3.30310900
183	C	5.39766100	0.52326800	-1.52714900
184	H	5.20434000	0.55610400	-0.45284300
185	H	5.17272400	-0.48784500	-1.87328600
186	H	6.44886200	0.76031200	-1.70999300
187	C	-4.44339200	-0.32255300	0.89131700
188	H	-5.17036700	-1.10620100	0.68792400
189	H	-4.45357100	-0.07139400	1.94875900

190	C	-3.05327000	-0.69746100	0.41508200
191	H	-2.75637500	-1.56287200	1.02459700
192	H	-3.07550900	-1.03079900	-0.62722900
193	C	-1.98595900	0.38964600	0.58163900
194	H	-2.21555500	1.23814700	-0.07603300
195	H	-1.96607000	0.74378000	1.61910600
196	C	-0.59296100	-0.16644200	0.23451000
197	H	-0.64477200	-0.76159200	-0.68464700
198	H	-0.27075200	-0.83415100	1.03983300
199	C	0.42272900	0.97326300	0.04979300
200	H	0.22895500	1.76183500	0.78549600
201	H	0.30893400	1.41728900	-0.94424800
202	C	1.85845000	0.54804100	0.27865400
203	H	2.55871300	1.34308400	0.02109600
204	H	2.02954800	0.20358400	1.29533200
205	Br	2.33956400	-1.01458600	-0.88235700
206	Br	-5.21571200	1.28791200	-0.00214600

(4) Cartesian Coordinates of Optimized Structures G1⊂P2

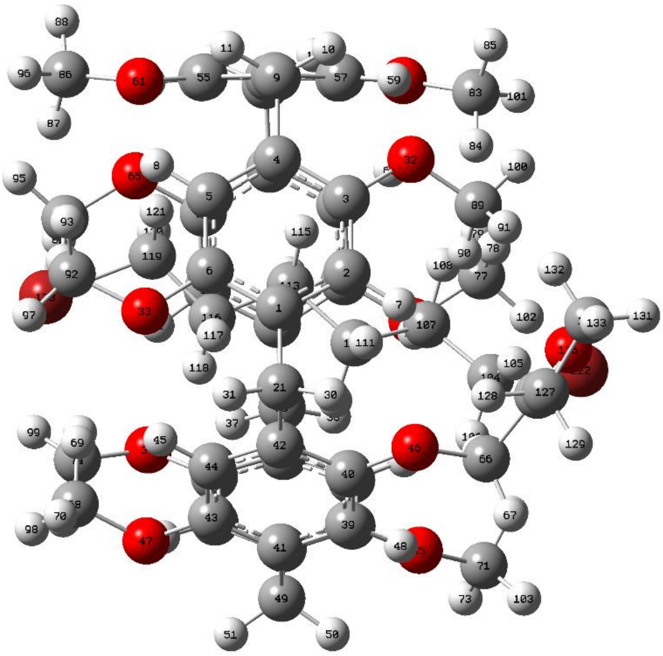

1	C	-1.15621500	-3.85159800	-0.06073000
2	C	-1.64969900	-3.18736400	-1.17890800
3	C	-0.83540600	-2.94716000	-2.28119400
4	C	0.48610100	-3.39815500	-2.27188400
5	C	1.00000300	-3.99220100	-1.12482100
6	C	0.19195300	-4.21113600	-0.01366100
7	H	-2.67978700	-2.85782300	-1.15253600
8	H	2.04900200	-4.25216100	-1.12616400
9	C	1.34769700	-3.20372400	-3.49920500
10	H	0.72783000	-3.30099200	-4.39237300
11	H	2.13090700	-3.96337700	-3.52096800
12	C	3.67643600	2.10219200	-3.12298800
13	H	3.43005500	2.71430700	-3.99220700

14	H	4.76061400	2.05842500	-3.00478700
15	C	3.05723800	2.69948700	-1.87931500
16	C	3.63537900	2.46906000	-0.63038000
17	C	1.86805100	3.41595600	-1.95368600
18	C	3.06913500	3.02909300	0.50655200
19	C	1.28540000	3.95175700	-0.81216600
20	C	1.91248700	3.79605500	0.42329600
21	C	-2.04992500	-4.15864500	1.11941200
22	C	-1.23022800	1.61128600	3.69398700
23	C	-1.78052100	2.48858800	2.76277200
24	C	0.14628800	1.61604900	3.90566100
25	C	-0.95891600	3.39802100	2.10003200
26	C	0.96351900	2.52248800	3.25048000
27	H	0.54591100	0.87039000	4.57603900
28	C	0.40537200	3.44039000	2.35429500
29	H	-1.35479100	4.06734900	1.34679800
30	H	-3.05599100	-4.39593000	0.77092400
31	H	-1.64509300	-5.01695900	1.65758800
32	O	-1.25524600	-2.29274200	-3.43052800
33	O	0.63309700	-4.79223500	1.16876000
34	O	2.33729600	2.59418300	3.43897100

35	O	-3.15757100	2.39173600	2.52316300
36	C	1.30711800	4.42894200	1.65447600
37	H	2.10189200	4.73490600	2.33808400
38	H	0.73106500	5.30555900	1.35452500
39	C	-3.10997100	-0.86856800	2.67496500
40	C	-3.09917700	-2.00410400	1.87676600
41	C	-2.12376700	-0.66703400	3.62977900
42	C	-2.09642000	-2.95597700	2.03039300
43	C	-1.09908500	-1.60697600	3.77072200
44	C	-1.09222900	-2.74149100	2.96813100
45	H	-0.29048200	-3.46576700	3.00533200
46	O	-4.04640900	-2.25606600	0.88071500
47	O	-0.12976600	-1.32838300	4.72252400
48	H	-3.84954600	-0.08967800	2.55082800
49	C	-2.09667500	0.61389200	4.42838800
50	H	-3.10685200	1.01524800	4.52470800
51	H	-1.68422000	0.42334900	5.42081100
52	C	3.11809300	0.71074900	-3.30113300
53	C	3.76006700	-0.38451900	-2.73861600
54	C	1.88542700	0.53510300	-3.93237500
55	C	3.19661800	-1.65186600	-2.80789100

56	H	4.67034500	-0.20061800	-2.18763200
57	C	1.32825200	-0.73392300	-4.00854400
58	C	1.98041700	-1.83411900	-3.46163700
59	H	0.35727700	-0.90329200	-4.44810100
60	O	1.28055000	1.67814000	-4.44079100
61	O	3.74679400	-2.77861200	-2.19703800
62	O	0.07784800	4.64104600	-0.80845100
63	H	1.39759700	3.49792000	-2.92169200
64	H	3.48058500	2.86217300	1.49020200
65	O	4.76491200	1.65204600	-0.60614200
66	C	-4.86140800	-1.13926000	0.44317800
67	H	-5.74821100	-1.02793100	1.07891700
68	C	1.01830200	-2.21450200	4.77813600
69	H	1.55313300	-2.23409600	3.82238600
70	H	0.72368500	-3.23300100	5.05548600
71	C	-3.84306400	3.64665600	2.21115200
72	H	-3.66277200	3.95226100	1.17697400
73	H	-3.52417500	4.43931300	2.89581500
74	C	2.96061100	1.52341100	4.19392500
75	H	2.77372200	0.54836600	3.72963000
76	H	2.61426500	1.50939000	5.23338600

77	C	-0.48576100	4.99089000	-2.10026000
78	H	-0.78899000	4.09773100	-2.65567400
79	H	0.22553900	5.57569800	-2.69444400
80	C	5.45562700	1.51045400	0.66293600
81	H	4.83396800	0.99231200	1.40141000
82	H	5.76520800	2.48375400	1.05973100
83	C	-0.08088500	1.55127200	-4.91750000
84	H	-0.74547000	1.18279200	-4.12777200
85	H	-0.14613000	0.88444300	-5.78472200
86	C	5.08078000	-2.64913900	-1.63964600
87	H	5.09200100	-1.97385800	-0.77701900
88	H	5.79365600	-2.29424500	-2.39217400
89	C	-2.45396300	-1.48369400	-3.32582700
90	H	-2.40473100	-0.81062600	-2.46416000
91	H	-3.35556100	-2.09347800	-3.24285800
92	C	2.03487600	-5.15486400	1.24934100
93	H	2.30464700	-5.88618000	0.47890100
94	H	2.67875200	-4.27282000	1.16385900
95	H	6.33354100	0.90306200	0.44313500
96	H	5.35281000	-3.65307400	-1.31431500
97	H	2.15763200	-5.60096300	2.23621500

98	H	1.66314500	-1.79726300	5.55171100
99	H	4.02854600	1.74077000	4.16801800
100	H	-2.49838600	-0.90307800	-4.24751700
101	H	-0.37990000	2.55861900	-5.20738400
102	H	-1.37190300	5.58163500	-1.87578700
103	H	-4.90481900	3.43744100	2.34435800
104	C	-2.97912000	1.69955100	-0.74486400
105	H	-3.55973800	0.89191600	-1.18886600
106	H	-3.19304600	1.80805700	0.31989000
107	C	-1.48429300	1.54734800	-0.97301900
108	H	-1.28208200	1.32437200	-2.02694200
109	H	-0.97075300	2.47853200	-0.71691000
110	C	-0.93727400	0.41739300	-0.07851800
111	H	-1.51209200	-0.50808800	-0.21606500
112	H	-1.04848600	0.71958600	0.96933200
113	C	0.54506600	0.13762400	-0.38245800
114	H	1.07588600	1.08667800	-0.52415200
115	H	0.63214200	-0.43662500	-1.31302600
116	C	1.20483900	-0.63127400	0.78004400
117	H	0.62871800	-1.53492200	1.00772500
118	H	1.20393200	0.01431600	1.66528500

119	C	2.61937400	-0.99538000	0.37525300
120	H	3.18654200	-0.13755700	0.01630900
121	H	2.64167800	-1.79799800	-0.35828900
122	Br	-3.70702100	3.34352200	-1.61685500
123	Br	3.68628200	-1.69844100	1.93029400
124	H	-4.29143300	-0.21024300	0.43955300
125	C	-5.24936200	-1.49381300	-0.98513000
126	O	-5.00598100	-0.73233200	-1.92406100
127	C	-5.90745700	-2.84149600	-1.14599200
128	H	-5.29958100	-3.56516700	-0.59379700
129	H	-6.87745700	-2.79358000	-0.63014800
130	C	-6.08988600	-3.23940200	-2.61225700
131	H	-6.62608600	-2.45936400	-3.15849900
132	H	-5.11749200	-3.37955200	-3.09264600
133	H	-6.64737400	-4.17639800	-2.68835000

References:

S1 Lin, Q.; Fan, Y.Q.; Mao, P.P.; Liu, L.; Liu, J.; Zhang, Y.M.; Yao, H.; Wei, T.B. Pillar[5]arene-based supramolecular organic framework with multi-guest detection and recyclable separation properties. *Chemistry* **2017**, *22*, 777-783.

S2 Du, X.S.; Wang, C.Y.; Jia, Q.; Deng, R.; Tian, H.S.; Zhang, H.Y.; Meguellati, K.; Yang, Y.W. Pillar[5]arene-based [1]rotaxane: high-yield synthesis, characterization and application in Knoevenagel reaction. *Chem. Commun*. **2017**, *53*, 5326-5329.

S3 Liu, L.Z.; Cao, D.R.; Jin, Y.; Tao, H.Q.; Kou, Y.H.; Meier, H. Efficient synthesis of copillar[5]arenes and their host-guest properties with dibromoalkanes. *Org. Biomol. Chem*. **2011**, *9*, 7007-7010.

S4 (a) Zhang, H.C.; Nguyen, K.T.; Ma, X.; Yan, H.; Guo, J.; Zhu, L.; Zhao, Y. Host-guest complexation driven dynamic supramolecular self-assembly. Org Biomol Chem. 2013, 11, 2070–2074. (b) Li, H.; Yang, Y.; Xu, F.F.; Liang, T.X; Wen, H.R.; Tian, W. Pillararene-based supramolecular polymers. *Chem. Commun*. **2019**, *55*, 271–285.
